# Host‐to‐Pathogen Transfer of Neutrophil Components via Extracellular Vesicles Shields *Candida albicans* From Immune Attack in Human Blood

**DOI:** 10.1002/jev2.70363

**Published:** 2026-08-29

**Authors:** Jennifer J. Patitz, Natalie E. Nieuwenhuizen, Anastasia Solomatina, Yann Bachelot, Thomas Krüger, Carl‐Magnus Svensson, Stephanie Hoeppener, Ann‐Kathrin Zimmermann, Matthew G. Blango, Ronny Martin, Olaf Kniemeyer, Theresa Lange, Axel A. Brakhage, Marc Thilo Figge, Oliver Kurzai, Kerstin Hünniger‐Ast

**Affiliations:** ^1^ Institute for Hygiene and Microbiology Julius Maximilians University of Würzburg Würzburg Germany; ^2^ Department of Applied Systems Biology Leibniz Institute for Natural Product Research and Infection Biology ‐ Hans Knöll Institute Jena Germany; ^3^ Faculty of Biological Sciences Friedrich Schiller University Jena Germany; ^4^ Department of Molecular and Applied Microbiology Leibniz Institute for Natural Product Research and Infection Biology ‐ Hans Knöll Institute Jena Germany; ^5^ Laboratory of Organic and Macromolecular Chemistry Friedrich Schiller University Jena Germany; ^6^ Jena Center for Soft Matter Friedrich Schiller University Jena Germany; ^7^ Junior Research Group RNA Biology of Fungal Infections Leibniz Institute for Natural Product Research and Infection Biology ‐ Hans Knöll Institute Jena Germany; ^8^ Department of Microbial Pathogenicity Mechanisms Leibniz Institute for Natural Product Research and Infection Biology ‐ Hans Knöll Institute Jena Germany; ^9^ Institute of Microbiology, Faculty of Biological Sciences Friedrich Schiller University Jena Germany; ^10^ Research Group Fungal Septomics Leibniz Institute for Natural Product Research and Infection Biology ‐ Hans Knöll Institute Jena Germany; ^11^ National Reference Center for Invasive Fungal Infections (NRZMyk) Leibniz Institute for Natural Product Research and Infection Biology ‐ Hans Knöll Institute Jena Germany

**Keywords:** bloodstream infection, *Candida albicans*, complement‐dependent opsonization, neutrophil‐derived extracellular vesicles (EVs), phagocytosis

## Abstract

Neutrophils effectively eliminate *Candida albicans* from human blood, but a subset of fungal cells escapes clearance and remains extracellular and viable. Here we show that this evasion is independent of known immune‐escape traits of *C. albicans*. Instead, neutrophil‐derived extracellular vesicles (EVs) enriched in antimicrobial proteins and neutrophil surface markers (CD66b, CD45, CD63, and complement receptors CR1, CR3 and CR4) promote this state. Isolated EVs bound to *C. albicans* preferentially in a complement‐dependent manner, and this binding was partially inhibited by anti‐CD11b, supporting CR3 involvement. Despite their antimicrobial cargo, EVs did not impair fungal growth. Instead, EV coating reduced neutrophil phagocytosis in purified‐cell and whole‐blood settings. These findings reveal a dual role for neutrophil‐derived EVs at the host‐pathogen interface: although enriched for innate effector molecules with potential antifungal activity, their deposition on *C. albicans* does not impair growth but is associated with reduced phagocytosis and maintenance of an extracellular population.

## Graphical abstract

Neutrophils release extracellular vesicles (EVs) in response to *Candida albicans*, and these EVs bind to the fungus in a complement‐dependent manner while carrying neutrophil surface markers and antimicrobial proteins. Despite this cargo, EV coating does not inhibit fungal growth but markedly reduces neutrophil phagocytic uptake in purified‐cell assays and whole blood. This reveals a host‐driven mechanism that promotes immune evasion and supports the maintenance of an extracellular *C. albicans* population during bloodstream infection.

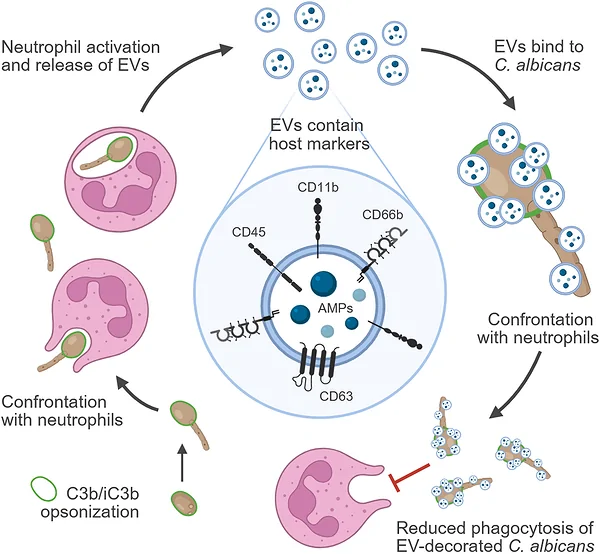

## 1 Introduction

*Candida albicans* is a major fungal pathogen and was recently classified as a critical priority fungal pathogen by the World Health Organization (WHO 2022). Each year, invasive candidiasis affects an estimated 1.5 million people worldwide, including around 600 000 bloodstream infections (BSI) with mortality rates of 30%–40% (Casalini et al. 2024; Cornely et al. 2025). These invasive infections mainly occur in hospitalized patients with underlying risk factors such as broad‐spectrum antibiotic treatment, central venous catheters, or total parenteral nutrition (Thomas‐Rüddel et al. 2022). Among host defense mechanisms, neutrophils are central to protection against *C. albicans*. Accordingly, neutropenia (< 500 neutrophils/mm^3^ blood) is a major risk factor for BSI and disseminated candidiasis (Duggan, Leonhardt, et al. 2015; Lass‐Flörl et al. 2024; Thomas‐Rüddel et al. 2022).

In human blood, *C. albicans* is predominantly phagocytosed by neutrophils (Hünniger et al. 2014), resulting in cytokine release and recruitment of other immune effector cells as well as effective fungal clearance (Duggan et al. 2015). Neutrophils control *C. albicans* through multiple mechanisms: phagocytosis, production of reactive oxygen and nitrogen species, release of antimicrobial granules, or formation of neutrophil extracellular traps (NETs) (Hünniger and Kurzai 2019). Among those, phagocytosis is considered the most effective mechanism for preventing hyphal growth and tissue invasion (Zhu et al. 2023). In contrast to other phagocytic cells, human neutrophils inhibit intracellular filamentation of *C. albicans* and efficiently kill engulfed fungal cells (Olivier et al. 2022; Vylkova and Lorenz 2014; Wozniok et al. 2008).

To counteract phagocytosis by neutrophils, *C. albicans* has evolved a variety of immune evasion strategies. Active remodeling of the fungal cell wall masks immunostimulatory pathogen‐associated molecular patterns (PAMPs) such as *β*‐1, 3‐D‐glucan beneath the outer mannan layer. This epitope masking is actively regulated by *C. albicans* in response to host environmental signals, including lactate, hypoxia, or iron limitation, and reduces recognition via pattern recognition receptors (PRRs) (Childers et al. 2020; Pradhan et al. 2019). *C. albicans* also interferes with complement‐mediated opsonization by expressing surface proteins that recruit host complement regulators such as factor H, C4‐binding protein and plasminogen. In addition, secreted proteases of the fungus may degrade complement proteins to prevent effective deposition of opsonins (C3b, iC3b) (Singh et al. 2020).

While all these strategies help *C. albicans* resist neutrophil activity, evade immune recognition, and enhance its potential for persistent infection, they rely on fungal metabolic activity or active remodeling of the fungal surface. However, previous work revealed that in human blood, a subpopulation of *C. albicans* cells consistently escapes phagocytosis and killing seemingly independent of any of the above‐mentioned mechanisms (Hünniger et al. 2014). Computational modeling indicated that the persistence of an extracellular *C. albicans* population depends on the dynamic interplay between fungal and host immune cells, and experimental evidence showed that this phenomenon is independent of fungal activity and even the viability of the affected fungal cell (Hünniger et al. 2014; Prausse et al. 2018).

Here, we demonstrate that this reduction in phagocytosis reflects a host‐driven mechanism mediated by neutrophil‐derived extracellular vesicles (EVs). These small membrane‐delimited particles associate with proteolytic enzymes, antimicrobial peptides, and other bioactive molecules, including RNA, and are known to modulate inflammation, regulate immune responses and mediate antimicrobial effects (Hong 2018; Hurtado Gutierrez et al. 2022). Neutrophil‐derived EVs decorate the surface of extracellular *C. albicans* cells with a variety of host‐cell markers. Surprisingly, instead of enhancing fungal clearance, this EV‐mediated coating hinders phagocytosis and promotes the maintenance of viable extracellular fungal cells in human blood. Our findings uncover a previously unrecognized mechanism whereby neutrophil‐derived EVs—typically considered part of the host defense—paradoxically reduce phagocytic uptake and facilitate persistence of an extracellular *C. albicans* population during bloodstream infection.

## 2 Materials and Methods

### 2.1 Ethics Statement

Human peripheral blood was collected from healthy volunteers with written informed consent. This study was conducted in accordance with the Declaration of Helsinki and all protocols were approved by the Ethics Committees of the University Hospital Jena (permit number: 3639–12/12) and the University Hospital Würzburg (permit number: 114/22).

### 2.2 *C. albicans* Strains and Culture

*C. albicans* wild‐type strain SC5314 (Gillum et al. 1984), BFP‐expressing (BFP^+^) *C. albicans* strain (ADH1/adh1::pADH1‐BFP‐SAT1‐ADH1t) (Duggan et al. 2015), GFP‐expressing (GFP^+^) *C. albicans cph1Δ/efg1Δ* mutant (see below), and BFP‐expressing (BFP^+^) *C. albicans cph1Δ/efg1Δ* mutant (see below) were cultivated on yeast extract‐peptone‐dextrose (YPD: 2% D‐glucose, 1% peptone, and 0.5% yeast extract in water) agar plates. For experiments, *C. albicans* cells were cultured overnight at 30°C in YPD medium. The following day, cells were reseeded in fresh YPD medium and grown at 30°C to mid‐log phase. Cells were then harvested and washed with Hank's Balanced Salt Solution (HBSS). To inactivate the cells, they were incubated in 0.1% thimerosal (Sigma‐Aldrich) in HBSS for 60 min at 37°C, followed by extensive washing to remove residual thimerosal. For opsonization, fungal cells were incubated in RPMI‐1640 medium supplemented with 5% autologous serum for 10 min at 37°C with shaking at 1000 rpm.

For construction of the GFP^+^ *C. albicans* *cph1Δ/efg1Δ* mutant strain (*cph1::hisG/cph1::hisG, efg1::hisG/efg1::hisG‐URA3‐hisG, ADH1/adh1::pADH1‐GFP‐SAT1‐ADH1t*) and BFP^+^ *C. albicans* *cph1Δ/efg1Δ* mutant strain (*cph1::hisG/cph1::hisG, efg1::hisG/efg1::hisG‐URA3‐hisG, ADH1/adh1::pADH1‐BFP‐SAT1‐ADH1t*) we excised either GFP or BFP cassettes containing ADH1 homology regions and SAT1 as selection marker with AscI and SacI from the plasmids pSK‐ADH1prom‐CaGFP‐SAT1 or pSK‐ADH1prom‐BFP‐SAT1 as previously described (Duggan et al. 2015; Hünniger et al. 2014). The cassettes were integrated into the *CaADH1* locus of *C. albicans* *cph1Δ/efg1Δ* mutant (provided by B. Hube, Leibniz‐HKI, Jena, Germany), using the lithium acetate transformation protocol (Walther and Wendland 2003). Transformants were grown for two days on YPD agar plates with 200 µg/mL of nourseothricine and verified by polymerase chain reaction (PCR) and microscopy.

### 2.3 *Ex Vivo* Whole‐Blood Infection Assay

Peripheral blood from healthy volunteers was collected in S‐Monovettes containing recombinant hirudin as an anticoagulant (Sarstedt). For infection assays, *C. albicans* cells (live and thimerosal‐killed BFP^+^ *C. albicans*, live BFP^+^ *cph1Δ/efg1Δ* mutant, as indicated) were added to whole blood at a concentration of 1 × 10^6^ cells/mL and incubated at 37°C under gentle rotation (5 rpm) for the specified time points. Following incubation, samples were immediately processed for flow cytometric analysis.

### 2.4 Cell Sorting of Non‐Phagocytosed *C. albicans* from Whole‐Blood Infection Samples

Whole blood infected for 120 min with live or thimerosal‐killed GFP^+^ *cph1Δ/efg1Δ* mutant cells was used for these experiments. The filamentation‐defective mutant was specifically chosen to facilitate flow cytometric cell sorting, as the absence of hyphal growth prevents technical complications associated with large filamentous structures. Samples were stained with mouse anti‐human CD45 (clone 2D1, BD Biosciences) for 20 min at 4°C, followed by treatment with cold ACK lysis buffer (Life Technologies) to remove erythrocytes. After washing with HBSS, cells were resuspended in HBSS and subjected to cell sorting using a BD FACSAria^TM^ II flow cytometry cell sorter (BD Bioscience) to isolate non‐phagocytosed *C. albicans* cells (GFP^+^/CD45^low^). Sorted *C. albicans* cells were subsequently stained with Calcofluor White (CFW, Sigma‐Aldrich) and propidium iodide (PI, Sigma‐Aldrich), fixed in ROTI Histofix 4% formaldehyde (Carl Roth), and centrifuged onto microscopic slides using a Cytospin device (300 x g for 5 min) for microscopic analysis using a LSM 780 confocal microscope (ZEISS). Image data were processed using ZEN 2012 software (ZEISS).

### 2.5 Isolation of Primary Human Neutrophils

Peripheral blood of healthy volunteers was collected in EDTA S‐Monovettes (Sarstedt). Untouched neutrophils were isolated from whole blood using the MACSxpress Whole Blood Neutrophil Isolation Kit (Miltenyi Biotec) according to the manufacturer's instructions. Red blood cell lysis was performed twice using cold ACK lysis buffer (Life Technologies). Isolated neutrophils were resuspended in RPMI‐1640 medium supplemented with 5% heat‐inactivated (60 min at 56°C), exosome‐depleted FBS (Gibco). Cell viability and concentration were assessed using a LUNA^TM^ cell counter (Logos Biosystems). After adjusting the cell concentration to 2 × 10^6^ cells/mL, the isolated cells were allowed to rest for 30 min at room temperature before infection. Cell purity was determined by flow cytometry using fluorochrome‐conjugated anti‐human antibodies against CD66b (clone REA306), CD45 (clone REA747), CD14 (clone REA599), CD3 (clone REAL104), and CD61 (clone REA761), all obtained from Miltenyi Biotec. Only cells with viability and purity greater than 95% were used for subsequent experiments.

### 2.6 Neutrophil Lysate Preparation

Isolated neutrophils (2 × 10^6^) were pelleted by centrifugation (300 x g, 5 min), and the supernatant was carefully aspirated. Pellets were lysed in ice‐cold RIPA buffer supplemented with protease inhibitor cocktail (both obtained from Sigma‐Aldrich) and incubated on ice for 10 min with periodic mixing to ensure complete solubilization. Lysates were clarified by centrifugation at 14,000 x g for 10 min at 4°C and the supernatant was collected for downstream analysis. Protein concentration was determined using a bicinchoninic acid (BCA) assay (Pierce). Equal amounts (40 µg) of protein were mixed with Tricine Sample Buffer (Bio‐Rad) containing reducing agent (β‐mercaptoethanol), heated at 70°C for 10 min and stored at −20°C for SDS‐PAGE / Western Blot analysis.

### 2.7 Primary Neutrophil Confrontation Assay

For confrontation experiments, neutrophils were either incubated with opsonized BFP^+^ *C. albicans*, opsonized BFP^+^ *cph1Δ/efg1Δ* mutant, or with HBSS as a mock‐infected control. Fungal cells were added to neutrophils at a multiplicity of infection (MOI) of 0.5. Samples were incubated for up to 60 min at 37°C under gentle rotation (5 rpm). Fungal cells incubated in medium without neutrophils served as control. Following incubation, samples were immediately processed for flow cytometric analysis or EV isolation.

The apoptotic fate of neutrophils was determined after incubation using the Annexin V‐FITC Kit (Miltenyi Biotec), followed by analysis via flow cytometry. Only confrontation samples exhibiting ≤ 10% apoptotic cells after 60 min were selected for EV isolation.

### 2.8 Isolation of EVs

EVs were isolated from 8 × 10^6^ neutrophils following 60 min confrontation with opsonized *C. albicans* (CaEVs), opsonized *cph1Δ/efg1Δ* mutant (cph1Δ/efg1ΔEVs), or from mock‐infected controls (SponEVs), using filtration and centrifugation protocols as previously described by (Shopova et al. 2020). Confrontation samples were centrifuged for 10 min at 1,000 x g and 4°C, and the supernatants were filtered by gravity using sterile polyvinylidene difluoride (PVDF) syringe filters (5.0 µm, Millex, Merck‐Millipore). The filtrates were subsequently centrifuged for 20 min at 19,500 x g and 4°C and supernatants were discarded. The resulting EV pellets were resuspended in RPMI‐1640 medium supplemented with 5% heat‐inactivated, exosome‐depleted FBS for downstream incubations, washed and resuspended in PBS (pH 7.4, Gibco) for nanoparticle tracking (NTA) and proteomic analysis or, alternatively, washed and resuspended in 50 mM ammonium acetate buffer for cryogenic transmission electron microscopy (cryo‐TEM). In selected experiments, isolated EVs or HBSS alone (dye‐only control) were incubated with MemGlow^TM^ 488 (MG488; Cytoskeleton, Inc.) for 10 min at room temperature. Both preparations were subsequently subjected to the identical centrifugation and washing procedure (20 min at 19,500 x g, 4°C) to remove unbound dye before resuspension in RPMI‐1640 medium supplemented with 5% heat‐inactivated, exosome‐depleted FBS.

### 2.9 Nanoparticle Tracking Analysis (NTA) of Isolated EVs

Particle size and concentration of EVs were analyzed using a NanoSight NS300 (Malvern Panalytical) equipped with a 642 nm laser at 24°C. Particle size distribution was determined from six technical replicates, and average values were calculated using the NanoSight NSXplorer software.

### 2.10 Cryogenic Transmission Electron Microscopy (Cryo‐TEM)

Cryo‐TEM imaging was performed on a Titan Krios G4 system at an acceleration voltage of 300 kV. Images were acquired either using a Ceta CMOS camera or with the Falcon IV direct electron detector. Samples were prepared by plunge freezing 9 µL of the solution onto precleaned Quantifoil grids (R2/2, Quantifoil). The grids were hydrophilized by plasma cleaning (Pelco Easy Glow, Plano) for 30 seconds. Plunge freezing was performed utilizing a Vitrobot Mark IV (FEI, Netherlands; plotting time 1 second, blot force ‐1) and liquid ethane as cryogen. During transfer to Autogrids and into the TEM the temperature was maintained always at a temperature below −160°C. Images were analyzed utiling the Velox software (Thermo Fisher Scientific).

### 2.11 SDS‐PAGE and Western Blot Analysis of CaEV Lysates

Following isolation, EVs derived from 8 × 10^6^ neutrophils challenged with opsonized *C. albicans* at an MOI of 0.5 were washed once with phosphate‐buffered saline (PBS, without Ca^2+^/Mg^2+^) and re‐pelleted under the same centrifugation conditions used for the final EV collection. For the detection of CD11b, CD63, Annexin 1 (ANXA1), and GAPDH, the washed EV pellet was lysed in ice‐cold RIPA buffer supplemented with protease inhibitor cocktail (both obtained from Sigma‐Aldrich) and incubated on ice for 10 min with periodic mixing to ensure complete solubilization. Lysates were clarified by centrifugation at 14,000 x g for 10 min at 4°C and the supernatant was collected for downstream analysis. The total EV lysate was mixed with 4x Laemmli sample buffer (Bio‐Rad) containing reducing agent (β‐mercaptoethanol), heated at 70°C for 10 min, and resolved by SDS‐PAGE on 4%–20% Mini‐PROTEAN TGX Precast Protein Gels (Bio‐Rad). For the detection of Cytochrome c (CYCS) and Calnexin (CANX), the EV pellet was directly denatured in Tricine sample buffer (Bio‐Rad) at 70°C for 10 min and resolved by SDS‐PAGE on 16.5% Mini‐PROTEAN Tris‐Tricine gels (Bio‐Rad). Neutrophil cell lysates were loaded as controls. Proteins were transferred to PVDF membranes using wet transfer according to the manufacturer's instructions. Membranes were blocked in EveryBlot Blocking Buffer (Bio‐Rad) for 10 min at room temperature and then incubated with primary rabbit monoclonal antibodies against CD11b (clone D6X1N), CD63 (clone E1W3T), ANXA1 (clone D5V2T), GAPDH (clone 14C10), CYCS (clone D18C7) and CANX (clone C5C9), all diluted 1:1,000 in blocking buffer, overnight at 4°C. All primary antibodies were obtained from Cell Signaling Technology.

After washing in TBS‐T, membranes were incubated with HRP‐conjugated goat anti‐rabbit IgG (Cell Signaling Technology) diluted 1:1,500 in blocking buffer for 60 min at room temperature, washed extensively with TBS‐T, and developed using enhanced chemiluminescence (SuperSignal^TM^ West Pico PLUS Chemiluminescent Substrate, Thermo Fisher Scientific). Chemiluminescent signals were captured using the iBright 1500 digital imaging system (Invitrogen). Molecular weight markers were run in parallel on each gel to confirm expected band sizes (Precision Plus Protein Dual Color Standards, Bio‐Rad).

### 2.12 LC‐MS/MS‐Based Proteome Analysis of Isolated EVs

#### 2.12.1 In‐Solution Digest

EVs isolated from 8 × 10^6^ neutrophils, either mock‐infected or challenged with opsonized *C. albicans* at an MOI of 0.5, were delipidated according to the protein precipitation method of Wessel and Flügge (Wessel and Flugge 1984). EV proteins were resolubilized in 100 µL of 50 mM triethyl ammonium bicarbonate (TEAB) in 1:1 (vol/vol) trifluoroethanol‐water. For reduction of cysteine thiols, the solution was mixed with 10 mM tris (2‐carboxyethyl) phosphine and alkylated with 12.5 mM chloroacetamide at 70°C for 30 min in the dark. Proteins were digested for 18 h at 37°C with trypsin‐LysC mix (Promega) at a protein‐to‐protease ratio of 25:1. Tryptic peptides were first completely evaporated using a vacuum concentrator (Eppendorf) and then resolubilized in 0.05% (vol/vol) trifluoroacetic acid (TFA) in 2:98 (vol/vol) acetonitrile‐water. Finally, the samples were filtered through Ultrafree‐MC hydrophilic polytetrafluoroethylene (PTFE) membrane (0.2 µm pore size) spin filters (Millipore). The filtrate was transferred to HPLC vials and stored at −70°C until LC‐MS/MS measurement.

#### 2.12.2 LC‐MS/MS Analysis

LC‐MS/MS analysis was performed on an Ultimate 3000 nano RSLC system connected to an Orbitrap Exploris 480 mass spectrometer equipped with a FAIMS interface (both Thermo Fisher Scientific). Peptide trapping for 5 min on an Acclaim Pep Map 100 column (2 cm x 75 µm, 3 µm) at 5 µL/min was followed by separation on a µPACneo 110 column. Mobile phase gradient elution of eluent A (0.1% (v/v) formic acid in water) mixed with eluent B (0.1% (v/v) formic acid in 90/10 acetonitrile/water) was performed using the following gradient: 0 min at 4% B and 750 nL/min, 10 min at 7.5% B and 750 nL/min, 12 min at 8% B and 300 nL/min, 70 min at 25% B and 300 nL/min, 100 min at 50% B and 300 nL/min, 105 min at 96% B and 300 nL/min, 108–110 min at 96% B and 750 nL/min, 110.1–120 min at 4% B and 750 nL/min. Positively charged ions were generated at spray voltage of 2.2 kV using a stainless steel emitter attached to the Nanospray Flex Ion Source (Thermo Fisher Scientific). The quadrupole/orbitrap instrument was operated in Full MS / data‐dependent MS2 mode. Precursor ions were monitored at *m/z* 300–1200 at a resolution of 120,000 FWHM (full width at half maximum) using a maximum injection time (ITmax) of 50 ms and 300% normalized AGC (automatic gain control) target. Precursor ions with a charge state of z = 2–5 were filtered at an isolation width of *m/z* 4.0 amu for further fragmentation at 28% HCD collision energy. MS2 ions were scanned at 15,000 FWHM (ITmax = 40 ms, AGC = 200%). Each sample was measured in triplicate with a different compensation voltage (−48 V, −63 V, −78 V).

#### 2.12.3 Protein Database Search

Tandem mass spectra were searched against the UniProt proteome databases (2025/03/13) of *Homo sapiens* (https://www.uniprot.org/proteomes/UP000005640) using Proteome Discoverer (PD) 3.0 (Thermo) and the database search algorithms (threshold search engine scores in parenthesis) Chimerys (>2), Mascot 3.1 (>30) using fragment intensity predictions with the MS2PIP:HCD2021 model, Comet (>3), MSFragger 4.1 (>8), MS Amanda 3.0 (>300), Sequest HT (>3) with and without INFERYS Rescoring, and MS PepSearch (>600) using NIST Spectral Libraries (https://chemdata.nist.gov/dokuwiki/doku.php?id=eptide:lib:humanhcd20160503). Two missed cleavages were allowed for the tryptic digestion. The precursor mass tolerance was set to 10 ppm and the fragment mass tolerance was set to 0.02 Da. Modifications were defined as dynamic Met oxidation, phosphorylation of Ser, Thr, and Tyr, protein N‐term acetylation with and without Met‐loss as well as static Cys carbamidomethylation. A strict false discovery rate (FDR) < 1% (peptide and protein level) was required for positive protein hits. The Percolator node of PD3.0 and a reverse decoy database was used for q‐value validation of spectral matches. Only rank 1 proteins and peptides of the top scored proteins were counted. Label‐free protein quantification was based on the Minora algorithm of PD3.0 using the precursor abundance based on intensity and a signal‐to‐noise ratio >10. Normalization was performed by using the total peptide amount method. Imputation of missing quan values was applied by using random abundance values between 50 and 100% of the lowest abundance identified. Differential protein abundance was defined as a fold change of > 2, *p*‐value < 0.05 and at least identified in 3 of 4 replicates of the sample group with the highest abundance. Statistics and data visualization were performed with R 4.4.2 and RStudio 2024.12.0.

To assess the presence of fungal proteins, the raw data were additionally searched against a combined UniProt database containing the *Homo sapiens* (UP000005640) and *Candida albicans* SC5314 (UP000000559) proteomes. Only 23 unique fungal peptides corresponding to 11 fungal proteins were identified, compared with more than 12,000 human peptides, indicating that fungal proteins contributed only negligibly to the dataset and were therefore not considered further.

### 2.13 Data Availability

The mass spectrometry proteomics data have been deposited at the ProteomeXchange Consortium via the PRIDE (Perez‐Riverol et al. 2025) partner repository with the dataset identifier PXD070460 and https://doi.org/10.6019/PXD070460.

### 2.14 Functional Annotation of the EV Proteome

Further analyses were based on the list of proteins identified from the database search (Supplemental Data 1). A prefiltering step was applied before comparing the protein sets from CaEVs and SponEVs. Albumin, keratin and hemoglobin proteins were excluded from the dataset, and only proteins detected in at least three out of four biological replicates were included in subsequent analyses.

Overlap analysis was performed using the webtool at https://bioinfogp.cnb.csic.es/tools/venny/index.html. REACTOME enrichment analysis was performed with gProfiler webtool (https://biit.cs.ut.ee/gprofiler/gost). The gene list was matched against *Homo sapiens*, and the significance threshold was corrected using Benjamini‐Hochberg FDR with a *p*‐value threshold of 0.05. Platelet‐related pathways (e.g. Platelet activation, signaling and aggregation) were considered likely due to platelet contamination of the neutrophil preparations and were therefore not highlighted in the main figure but are listed in Supplemental Data 2.

### 2.15 MPO Activity Assay

Myeloperoxidase (MPO) activity was determined using the TMB High Sensitivity Substrate Solution (BioLegend) in a kinetic colorimetric assay. Neutrophils (1 × 10^4^ cells per sample) were resuspended either in HBSS or in 0.05% Triton X‐100 and incubated for 10 min at room temperature to achieve cell lysis. Where indicated, samples were subsequently incubated with the selective MPO inhibitor 4‐aminobenzoic acid hydrazide (4‐ABAH, 100 µM, Sigma‐Aldrich) for 15 min at 37°C. TMB High Sensitivity Substrate Solution was then added at a 1:1 (v/v) ratio, and absorbance at 650 nm was recorded kinetically every 5 min at 37°C using a microplate reader (Tecan). CaEVs were isolated from co‐incubations of 1.6 × 10^7^ neutrophils with 8 × 10^6^ opsonized *C. albicans* for 60 min (MOI = 0.5), as described above. Isolated CaEVs were either resuspended in HBSS or lysed with 0.05% Triton X‐100 for 10 min at room temperature before addition of TMB High Sensitivity Substrate Solution (1:1, v/v). MPO activity was monitored kinetically under the same conditions as described for neutrophils. For quantitative analysis, OD_650_ values corresponding to the maximal signal were used for neutrophils and CaEVs, respectively.

### 2.16 Incubation of *C. albicans* with Isolated EVs

CaEVs isolated from supernatants of neutrophil‐*C. albicans* confrontations (8 × 10^6^ neutrophils and 4 × 10^6^ *C. albicans*, MOI = 0.5) were pooled and incubated with 1 × 10^5^ BFP^+^ *C. albicans* (opsonized or non‐opsonized, live or thimerosal‐killed, as indicated) for 60 min at 37°C. As a control, fungal cells were incubated in medium without EVs (no EVs). These control samples were subjected to the same staining procedures as EV‐treated samples and served to assess potential background signals arising from the opsonization process.

In selected experiments, aliquots of CaEVs and medium without EVs were pre‐incubated with BD Pharmingen purified NA/LE mouse anti‐human CD11b (clone ICRF44) or the corresponding isotype control (BD Pharmingen purified NA/LE mouse IgG1, clone 107.3), both from BD Biosciences, for 10 min at 37°C. Following incubation, samples were immediately processed for flow cytometric analysis.

For imaging flow cytometry, MG488‐stained CaEVs were incubated with 1 × 10^5^ opsonized or non‐opsonized live *C. albicans* for 60 min at 37°C and subsequently processed immediately. To provide independent high‐resolution confirmation of EV association with the fungal surface, MG488‐labelled CaEVs were additionally incubated with 1×10^5^ opsonized live *C. albicans* cells in µ‐Slide 8‐well chambers (ibidi) for 60 min at 37°C. EV binding was visualized using a LSM 780 laser scanning confocal microscope (ZEISS) equipped with a Plan‐Apochromat 63x/1.4 oil immersion objective. Z‐stack images were acquired and processed as maximum intensity projections using ZEN 2012 software (ZEISS).

### 2.17 Live Cell Imaging of Fungal Growth in the Presence of Isolated CaEVs

Opsonized *C. albicans* (2 × 10^4^ cells) were seeded into µ‐Slide 8‐well chambers (ibidi) and incubated with isolated CaEVs or in medium without EVs (no EVs) as a control in a total volume of 200 µL per well. Assays were maintained in an environmental control chamber at 37°C and 5% CO_2_. Time‐lapse images were acquired every 5 min over a period of 10 h using a LSM 780 confocal microscope (ZEISS), focusing on the bottom plane of the imaging chamber. Fungal growth was monitored with a Plan‐Apochromat 20x/0.8 objective and image data were processed using ZEN 2012 software (ZEISS).

### 2.18 Analysis of Live Cell Imaging

The image analysis pipeline was implemented in JIPipe (Gerst et al. 2023), and the code is available at https://github.com/applied‐systems‐biology/C‐albicans‐hyphal‐growth. Yeast and hyphal forms of *C. albicans* were segmented using Canny edge detection (Canny 1986), followed by morphological closing and opening operations to connect fragmented segments and remove small artifacts. The segmented hyphae were then skeletonized, and hyphal length was quantified using the native JIPipe filament analysis module. To account for differences in the number of yeast cells in each time series, the total hyphal length per frame was normalized to the initial number of yeast cells.

### 2.19 Phagocytosis of EV‐Treated *C. albicans*

Phagocytosis of *C. albicans* cells pre‐incubated with CaEVs was analyzed using flow cytometry. Opsonized thimerosal‐killed BFP^+^ *C. albicans* cells were incubated with isolated CaEVs for 60 min at 37°C to generate EV‐treated *C. albicans* (^EV^ *C.a*.). As a control, *C. albicans* cells were incubated in medium without EVs (*C.a*.). The samples were centrifuged and pelleted fungal cells were resuspended in medium containing freshly isolated neutrophils at an MOI of 0.5 (4 × 10^5^ *C. albicans* and 8 × 10^5^ neutrophils in 500 µL), followed by incubation for 30 min at 37°C under gentle rotation (5 rpm). Neutrophils were isolated from peripheral blood obtained from the same donor used for the initial CaEV generation. Phagocytosis of EV‐treated *C. albicans* during whole‐blood infection was performed in a similar manner. Hirudin‐anticoagulated whole blood (500 µL), obtained from the same donor, was infected with ^EV^ *C.a*. and *C.a*. (4 × 10^5^ each) and incubated for 60 min at 37°C under gentle rotation (5 rpm). Following incubation, samples were immediately processed for flow cytometric analysis.

### 2.20 Flow Cytometry

Differential staining and flow cytometry were employed to analyze distinct immune cell populations in whole‐blood samples with respect to their association with *C. albicans*, and to investigate the presence of markers on extracellular *C. albicans* cells. Whole blood was stained with fluorochrome‐conjugated anti‐human CD66b (clone REA306) and CD14 (clone REA599), both from Miltenyi Biotec. Following incubation for 20 min at 4°C, erythrocytes were lysed with BD FACS Lysing solution, followed by washing and harvesting cells in BD CellWASH solution (both BD Biosciences). Changes in surface expression on extracellular *C. albicans* cells during neutrophil confrontation and on *C. albicans* cells following incubation with isolated CaEVs were analyzed using fluorochrome‐conjugated anti‐human antibodies against CD66b (clone REA306), CD11b (clone REA713), CD45 (clone REA747), CD63 (clone REA1055), and CD88 (clone REA1213), all obtained from Miltenyi Biotec. In parallel, staining with the appropriate isotype controls (REA Control Antibody (S), human IgG1 [rIgG1], clone REA293, Miltenyi Biotec) was included to assess binding specificity. Staining of fungal cells incubated in medium alone (without neutrophils or EVs) was performed in the same manner and served as a control. Following incubation for 20 min at 4°C, samples were immediately analyzed by flow cytometry. C3b/iC3b opsonization on fungal cells was analyzed using a mouse anti‐human C3/C3b/iC3b antibody (clone 6C9; ImmunoTools) and a mouse IgG1 isotype control (clone 203, ImmunoTools). Analysis of Annexin V binding to *C. albicans* cells was performed using Annexin V‐FITC (Miltenyi Biotec) according to the manufacturer's instructions.

Data acquisition was performed using either the BD FACSCanto^TM^ II flow cytometer with BD FACSDiva v6.1.3 software (both BD Bioscience) or the MACSQuant X flow cytometer with MACSQuantify v2.11 software (both Miltenyi Biotec). FlowJo v10.10.0 or FlowLogic v8.7 software was used for analysis. The strategy used to assess the association of *C. albicans* with neutrophils and monocytes in human blood was adapted from (Hünniger et al. 2014), and used in the same way for BFP^+^ *C. albicans* cells in this study. As previous studies demonstrated no detectable association between *C. albicans* and lymphocytes, regardless of fungal viability (Hünniger et al. 2014; Lehnert et al. 2021), we did not analyze lymphocytes in the present study. The gating strategy used to identify non‐phagocytosed (extracellular) *C. albicans* cells during neutrophil confrontation is shown in Figure S1.

### 2.21 Imaging Flow Cytometry

Whole blood and neutrophil confrontation samples were stained with fluorochrome‐conjugated anti‐human CD45 (clone REA747, Miltenyi Biotec) and CD66b (clone REA306, Miltenyi Biotec), and processed as described for flow cytometry. In parallel, appropriate isotype controls were included in all analyses to assess binding specificity, using REA Control Antibody (S), human IgG1 (rIgG1, clone REA293, Miltenyi Biotec). *C. albicans* cells incubated with MG488‐stained EVs were analyzed directly following incubation. Data acquisition was performed using the Cytek Amnis ImageStream X Mk II Imaging Flow Cytometer (Cytek) at 60 x magnification. Bright field (BF) images were captured in channels 1 and 9, MG488 in channel 2, PerCP‐Vio 700 in channel 5, PE‐Vio 770 in channel 6, BFP in channel 7 and side scatter (SSC) in channel 12. All events, including the instrument's internal speed beads, were recorded. Data analysis was performed using the Image Data Exploration and Analysis Software (IDEAS 6.2, Amnis).

Extracellular BFP^+^ *C. albicans* from whole blood and neutrophil infection samples were identified as BFP^+^/CD45^low^ events and subsequently analyzed for the presence of surface CD66b and MG488 signals.

### 2.22 Statistical Analysis

Statistics and data visualization were performed with R4.3.3, RStudio 2024.12.0 and GraphPad Prism 10. FlowJo v10.10.0 or FlowLogic v8.7 software was used for analysis of flow cytometry data.

For all experiments, at least three independent replicates were performed using cells from different donors. Data are presented as arithmetic means ± standard deviation (SD). In most cases, statistical significance was determined using a two‐tailed unpaired or paired *t‐*test or two‐way ANOVA (as indicated). Significance levels are indicated as follows: * *p* < 0.05, ** *p* < 0.01, *** *p* < 0.001, and **** *p* < 0.0001. Statistical analyses of proteome data are described in section ‘Protein data base search’.

### 2.23 Mathematical Modeling

A previously established state‐based virtual infection model (Hünniger et al. 2014), which has also been applied to investigate host‐pathogen interactions and phagocytosis kinetics in whole‐blood infection assays (Lehnert, Prausse, et al. 2021; Timme et al. 2018), was used to describe the interactions between *C. albicans* and immune cells in whole blood. The model includes pathogen populations in distinct states—extracellular, alive, killed, associated with neutrophils or monocytes—with transitions governed by rate parameters for phagocytosis ($\phi_{N}$ for neutrophils, $\phi_{M}$ for monocytes), killing ($\kappa_{N}$ for neutrophils, $\kappa_{M}$ for monocytes, $\kappa_{EK}$ and γ for extracellular killing), and immune evasion (ρ). The implementation of the model and the analysis scripts are available at https://github.com/applied‐systems‐biology/SBM_wholeblood_EVdecoration.

To assess the impact of extracellular vesicle deposition, distinct phagocytosis rates were introduced for EV‐decorated *C. albicans* $\phi_{N}$(^EV^ *C.a*.) and untreated control cells $\phi_{N}$(*C.a*.). Parameter inference was performed using Approximate Bayesian Computation implemented via the pyABC Python library (Schälte et al. 2022). Simulated population dynamics were compared with experimental data, and the discrepancy between them was quantified using a weighted mean squared error cost function defined as

$$d\;\left(x_{\mathrm{sim}},x_{\exp}\right)=\frac{1}{n_{p}}\;\sum_{j=1}^{n_{p}}\frac{w_{j}}{n_{t,j}}\sum_{i=1}^{n_{t,j}}{\left(x_{i,j,\mathrm{sim}}-x_{i,j,\exp}\right)}^{2},$$

where $x_{i,j,\mathrm{sim}}$ and $x_{i,j,\exp}$ denote the simulated and experimental values, respectively, for population j at time point i. The total number of populations and time points are given by $n_{p}$ and $n_{t,j}$. The population‐specific weights $w_{j}$ were defined as the inverse of the mean variance in the corresponding experimental population, thereby accounting for differences in measurement uncertainty across populations. Parameter sets with a cost $d(x_{\mathrm{sim}},x_{\exp})<\varepsilon =0.25$ were accepted to approximate the posterior distribution.

Final fits were obtained by averaging over 100 stochastic simulations using parameter sets randomly sampled from the posterior distribution. Parameter estimates correspond to the mode of the joint posterior distribution, which was used to quantify the differences in phagocytosis rates by neutrophils of EV‐decorated and untreated *C. albicans*.

## 3 Results

### 3.1 Extracellular *C. albicans* Cells in Blood Remain Viable and Acquire the Neutrophil Marker CD66b

In *ex vivo* human blood, *C. albicans* is rapidly phagocytosed by neutrophils and, to a lesser extent, by monocytes (Hünniger et al. 2014; Kämmer et al. 2020). However, a relevant subset of *C. albicans* cells (∼15%) can escape phagocytosis and persist extracellularly for hours (Hünniger et al. 2014). These findings suggest that *C. albicans* may resist efficient clearance *in vivo*, facilitating its dissemination and persistence in systemic infections. Importantly, the size of this escape subset mainly depends on neutrophil numbers and activity, which may contribute to the increased risk of disseminated infection observed in neutropenic patients (Desai and Lionakis 2018; Hope et al. 2007; Timme et al. 2018).

**FIGURE 1:**
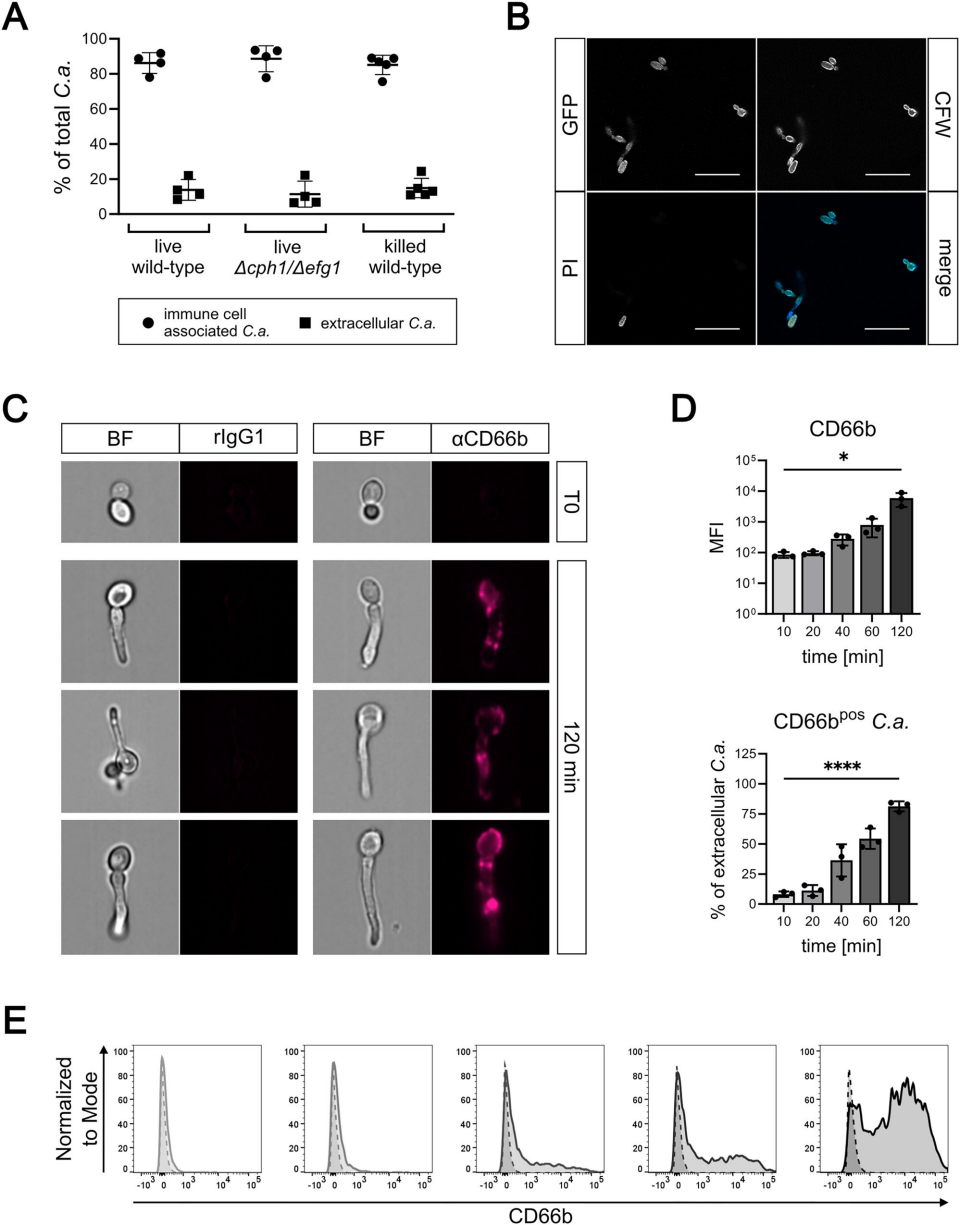
Extracellular *C. albicans* cells in whole blood remain viable and gain the neutrophil marker CD66b. (A) Distribution of live and thimerosal‐killed BFP^+^ *C. albicans* (wild‐type) and live BFP^+^ *cph1Δ/efg1Δ* mutant (120 min *p.i*.) as determined by flow cytometry. The percentages of *C. albicans* associated with neutrophils and monocytes (dots) and of extracellular fungal cells (squares) were calculated relative to total *C. albicans* cells in blood (set to 100%). All values correspond to the means and SD of at least four independent experiments with whole blood from different donors. (B) Cell sorting of extracellular live GFP^+^ *cph1Δ/efg1Δ* mutant cells after 120 min of whole‐blood infection and subsequent staining with calcofluor white (CFW) and propidium iodide (PI) revealed the majority of fungi to be alive. Representative fluorescence images were taken using a Zeiss LSM 780 confocal microscope. In the merged image, GFP fluorescence appears green, CFW staining blue, and PI staining red. Scale: 20 µm. The non‐filamentous *cph1Δ/efg1Δ* mutant was used for technical reasons related to cell sorting. (C) Visualization of the neutrophil marker CD66b on extracellular *C. albicans* after 120 min of whole‐blood infection acquired using imaging flow cytometry with fluorochrome‐conjugated αCD66b antibody or the appropriate isotype control (rIgG1). Analysis of *C. albicans* cells in blood at time point 0 (T0) served as a control for the baseline condition. Data are representative of three experiments using blood from different donors (BF—Bright Field). (D) Quantitative analysis of CD66b expression on the surface of extracellular BFP^+^ *C. albicans* cells, based on median fluorescence intensity (MFI, top panel), and of the proportion of CD66b‐positive (CD66b^pos^) cells among the total population of extracellular fungal cells (bottom panel) at different time points during human whole‐blood infection (mean ± SD). Statistical analysis was performed using the two‐tailed unpaired *t*‐test, * *p* < 0.05, **** *p* < 0.0001. (E) Related histograms show the isotype control (dashed line) and antibody staining (solid line) and are representative of three independent experiments using blood from different donors.

In our previous whole‐blood studies, association of *C. albicans* with immune cells was monitored for up to 240 min, but no substantial changes in fungal distribution, particularly within the extracellular population, were observed beyond 120 min (Hünniger et al. 2014). Therefore, the present experiments focused on the 120 min time point, which adequately captures the relevant host‐pathogen interactions. To determine whether fungal escape depends on viability or morphology, we performed whole‐blood infection assays using thimerosal‐killed wild‐type cells and live non‐filamentous mutant cells (*cph1Δ/efg1Δ*). Both showed extracellular fungal levels comparable to those of live wild‐type *C. albicans* after 120 min (Figure 1A), indicating that persistence within the extracellular population in human blood is independent of filamentation or any active fungal response. Flow cytometric sorting of extracellular *C. albicans* combined with propidium iodide (PI) staining further revealed that most extracellular fungal cells remain viable, demonstrating that these cells escape not only phagocytosis but also extracellular killing mechanisms (Figure 1B, Figure S2). For these sorting experiments, the *cph1Δ/efg1Δ* mutant was used to avoid technical complications caused by large hyphal structures during flow cytometric cell sorting. These results align with our previous mathematical modeling predictions that a subpopulation of *C. albicans* escapes phagocytosis and extracellular killing in human blood independent of any fungal activity (Hünniger et al. 2014).

Further characterization of the extracellular *C. albicans* population using flow cytometry in combination with imaging unexpectedly revealed the prominent recruitment of the neutrophil marker CD66b to the surface of extracellular fungi (Figure 1C–E). We observed a progressive increase in both the overall level of CD66b on the fungal surface (*p* < 0.05) and the frequency of CD66b‐positivity within the extracellular *C. albicans* population (*p* < 0.0001) over time (Figure 1D,E). Based on this observation, we hypothesized that host‐mediated remodeling of the fungal surface contributes to the reduced phagocytic clearance and persistence of extracellular *C. albicans* in human blood.

### 3.2 Neutrophils Decorate *C. albicans* with Host‐Cell Molecules

**FIGURE 2:**
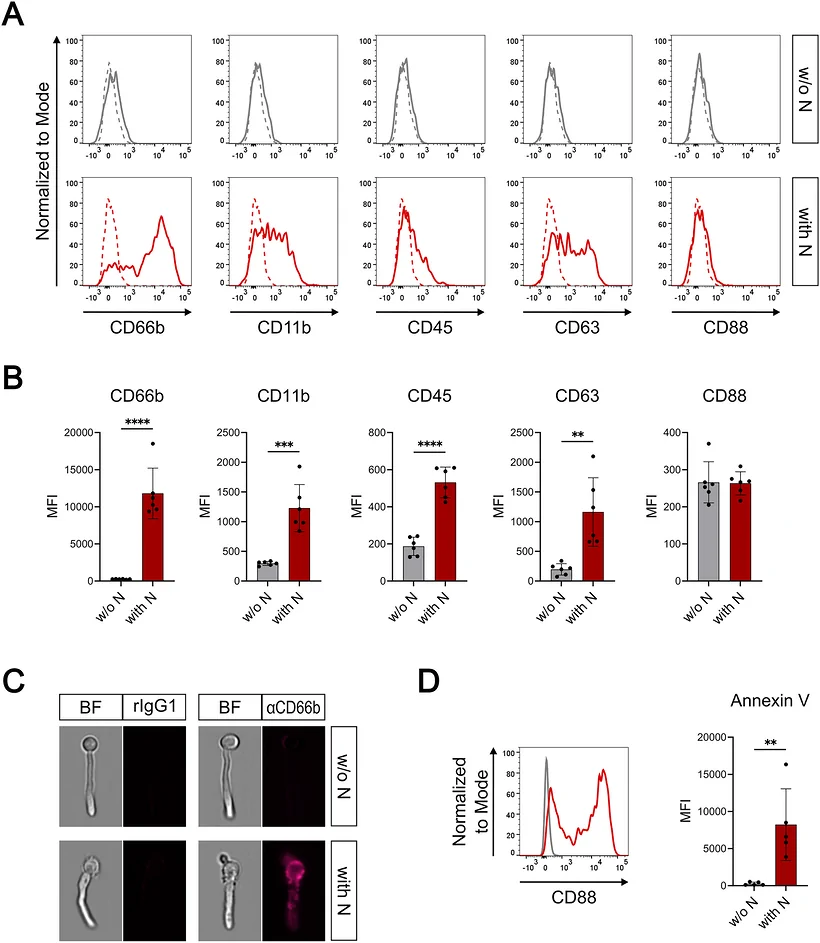
Deposition of neutrophil markers on extracellular *C. albicans* cells during neutrophil confrontation. Opsonized BFP^+^ *C. albicans* cells were confronted with human neutrophils for 60 min (with N). Extracellular fungal cells were analyzed by flow cytometry and compared to *C. albicans* incubated in medium without immune cells as a control (w/o N). (A) Histograms of host cell markers showing isotype control (dashed line) and antibody staining (solid line) for *C. albicans* incubated in medium (grey) or with neutrophils (red). Data are representative of six independent experiments using blood from different donors. (B) Quantitative analysis of CD66b, CD11b, CD45, CD63 and CD88 associated with extracellular *C. albicans*, based on median fluorescence intensity (MFI, mean ± SD from *n* = 6 experiments). (C) Visualization of the neutrophil marker CD66b on extracellular *C. albicans*, acquired using imaging flow cytometry with fluorochrome‐conjugated αCD66b antibody or the appropriate isotype control (rIgG1). Data are representative of four independent experiments (BF—Bright Field). (D) Annexin V staining of extracellular *C. albicans* cells during neutrophil confrontation. Representative histograms of Annexin V binding are shown for *C. albicans* incubated in medium (grey) and for extracellular *C. albicans* during neutrophil confrontation (red), and related quantitative analysis (mean ± SD from *n* = 5 experiments). Statistical analyses for B and D were performed using the two‐tailed unpaired *t*‐test, ** *p* < 0.01, *** *p* < 0.001, **** *p *< 0.0001.

To further characterize deposition of host molecules on the fungal surface, we analyzed the surface of extracellular *C. albicans* during *ex vivo* confrontation with purified human neutrophils. After 60 min of confrontation with neutrophils, CD66b, CD45, CD11b, CD63 but not CD88 were detected on the fungal surface, suggesting deposition of a broad range of neutrophil‐derived components (Figure 2A,B). Imaging flow cytometry showed CD66b localization as punctate structures on the surface of non‐phagocytosed *C. albicans* cells (Figure 2C), indistinguishable from the pattern observed before in whole blood (Figure 1C). In addition, extracellular *C. albicans* also showed positive Annexin V staining (Figure 2D). Annexin V is a protein that binds to phosphatidylserine exposed on the outer membrane of EVs and apoptotic bodies (Kolonics et al. 2020). Together, these findings suggested that EVs or apoptotic bodies might bind to the fungal surface. Therefore, we next assessed neutrophil viability during the interaction by performing a flow cytometry‐based apoptosis assay. Both mock‐infected and *C. albicans*‐infected neutrophils maintained viabilities of > 80% after 60 min of incubation and only ≈10% of cells were apoptotic (Figure S3). This argues against apoptotic body release as a major mechanism for deposition of neutrophil markers on the fungal surface. Taken together, these findings suggest that neutrophil‐derived EVs bind to *C. albicans* and result in deposition of neutrophil markers on the fungal surface.

### 3.3 Exposure to *C. albicans* Enhances EV Release by Neutrophils

**FIGURE 3:**
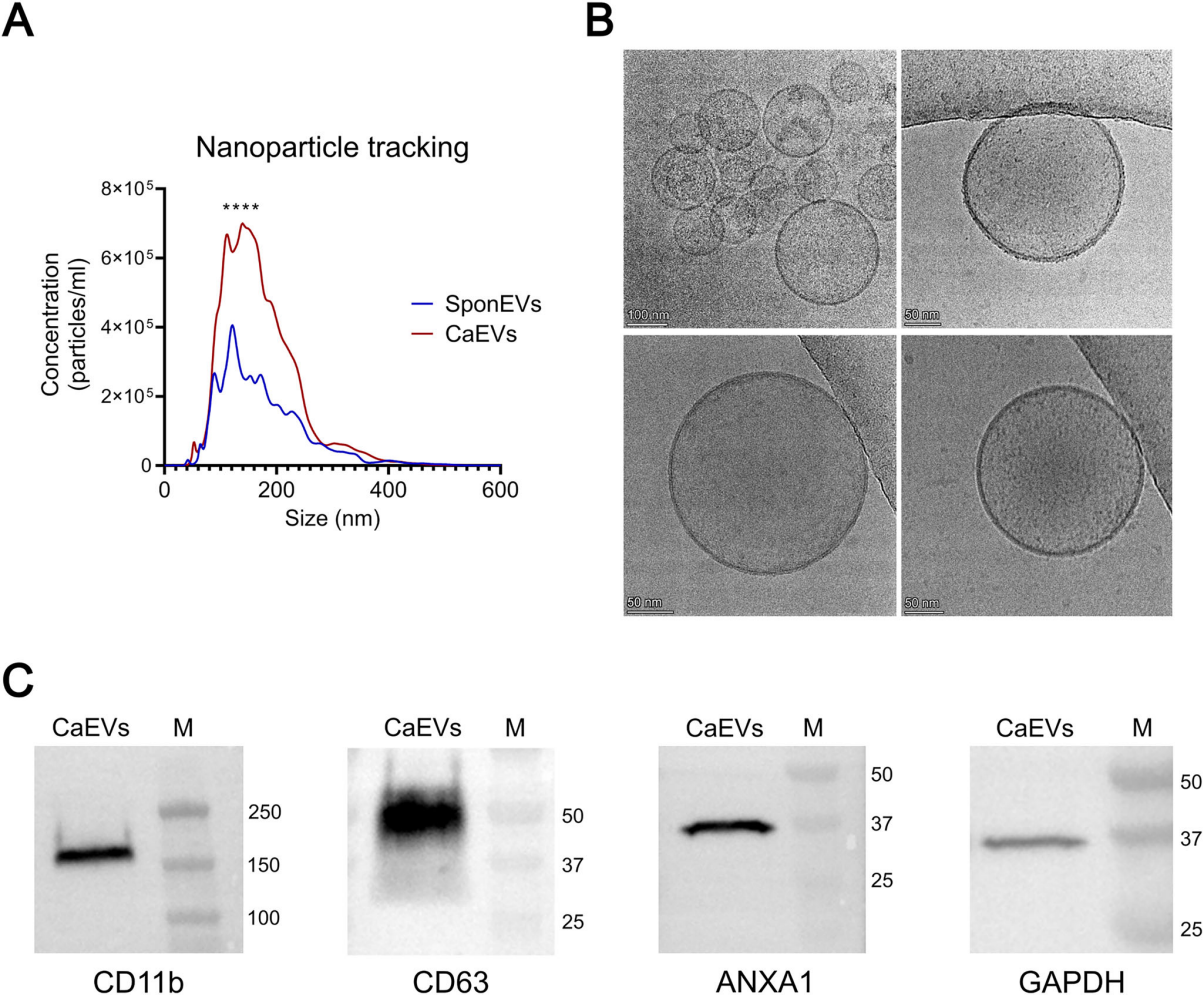
Characterization of neutrophil‐derived EVs. EVs were isolated from neutrophils confronted with opsonized *C. albicans* (CaEVs) or mock‐infected neutrophils (SponEVs) after 60 min of incubation. (A) Particle size and concentration of isolated EVs was determined using a NanoSight NS300. Statistical analysis was performed using two‐way ANOVA with Šídák post‐test, *n* = 5 biological replicates, **** *p* < 0.0001. (B) Cryogenic transmission electron microscopy (cryo‐TEM) images of CaEVs revealing predominantly spherical vesicles with well‐defined bilayer structures; scale bars as indicated (50–100 nm). (C) Representative immunoblot analyses of vesicle‐associated proteins (CD11b, CD63, ANXA1, and GAPDH) from CaEV preparations, with molecular weight markers (M) indicated.

EVs are released under both resting and activated conditions and contain protein cargo reflective of their cellular origin (Shopova et al. 2020; Zhou and Brechard 2022). To investigate whether confrontation with *C. albicans* triggers EV release by neutrophils, we isolated and characterized EVs from neutrophils exposed to opsonized *C. albicans* (CaEVs) and compared them to EVs from mock‐infected neutrophils (SponEVs). Nanoparticle tracking analysis (NTA) showed that CaEVs and SponEVs isolated 60 min post‐infection exhibited comparable size distributions, with predominant particle populations in the range of approximately 100–200 nm and mean diameters of approximately 140 nm and 120 nm, respectively (Figure 3A). However, CaEVs were released at significantly higher concentrations (9.27 × 10^7^ particles/mL) compared to SponEVs (5.11 × 10^7^ particles/mL, *p* < 0.0001).

To further assess vesicle morphology and structural integrity, CaEV preparations were analyzed by cryogenic transmission electron microscopy (cryo‐TEM) (Figure 3B). Cryo‐TEM was selected to minimize potential artifacts associated with negative staining and to avoid drying‐induced structural alterations. The acquired images revealed predominantly spherical, membrane‐enclosed particles with smooth contours and well‐preserved morphology. The vesicles exhibited a defined and intact membrane, and in some instances, the lipid bilayer double‐layer structure was distinctly visible. Vesicle diameters observed by cryo‐TEM were in good agreement with the size distribution determined by NTA. Importantly, no evidence of vesicle collapse, membrane disruption, multilamellar structures, or ice crystal artifacts was observed, supporting the presence of intact vesicles.

Biochemical characterization of CaEVs was performed in accordance with MISEV recommendations using Western blot analysis of EV lysates (Figure 3C). CaEV preparations were positive for the neutrophil‐associated protein CD11b (∼170 kDa) and for Annexin A1 (ANXA1, ∼38 kDa), an EV‐associated, vesicle‐related protein repeatedly reported to be enriched in neutrophil‐derived EVs. In addition, the EV‐associated tetraspanin CD63 (25–60 kDa) was detected. GAPDH (∼37 kDa), a cytosolic protein, was observed in CaEV preparations, consistent with previous reports indicating that certain intracellular proteins can be associated with EV fractions (Welsh et al. 2024). To assess the purity of EV preparations, we analysed the presence of the endoplasmic reticulum protein Calnexin (CANX) and the mitochondrial protein Cytochrome c (CYCS) (Figure S4). CANX was detected as a single major band at ∼90 kDa, corresponding to the full‐length glycosylated protein. An additional band at ∼65 kDa was observed selectively in neutrophil lysates and interpreted as a non‐specific or proteolytic signal. While CANX was robustly present in neutrophil lysates, only a barely detectable signal was observed in EV preparations. Similarly, CYCS (∼12 kDa) was readily detected in neutrophil lysates but was absent from EV samples. These findings indicate that EV preparations contain negligible levels of intracellular contaminants.

Together, the combined analysis of particle size and concentration (NTA), vesicle morphology (cryo‐TEM), and protein marker expression (Western blot) supports the conclusion that confrontation of neutrophils with *C. albicans* results in the enhanced release of EVs of neutrophil origin.

### 3.4 Fungal Challenge Drives Quantitative and Qualitative Changes in the Proteome of Neutrophil‐Derived EVs

**FIGURE 4:**
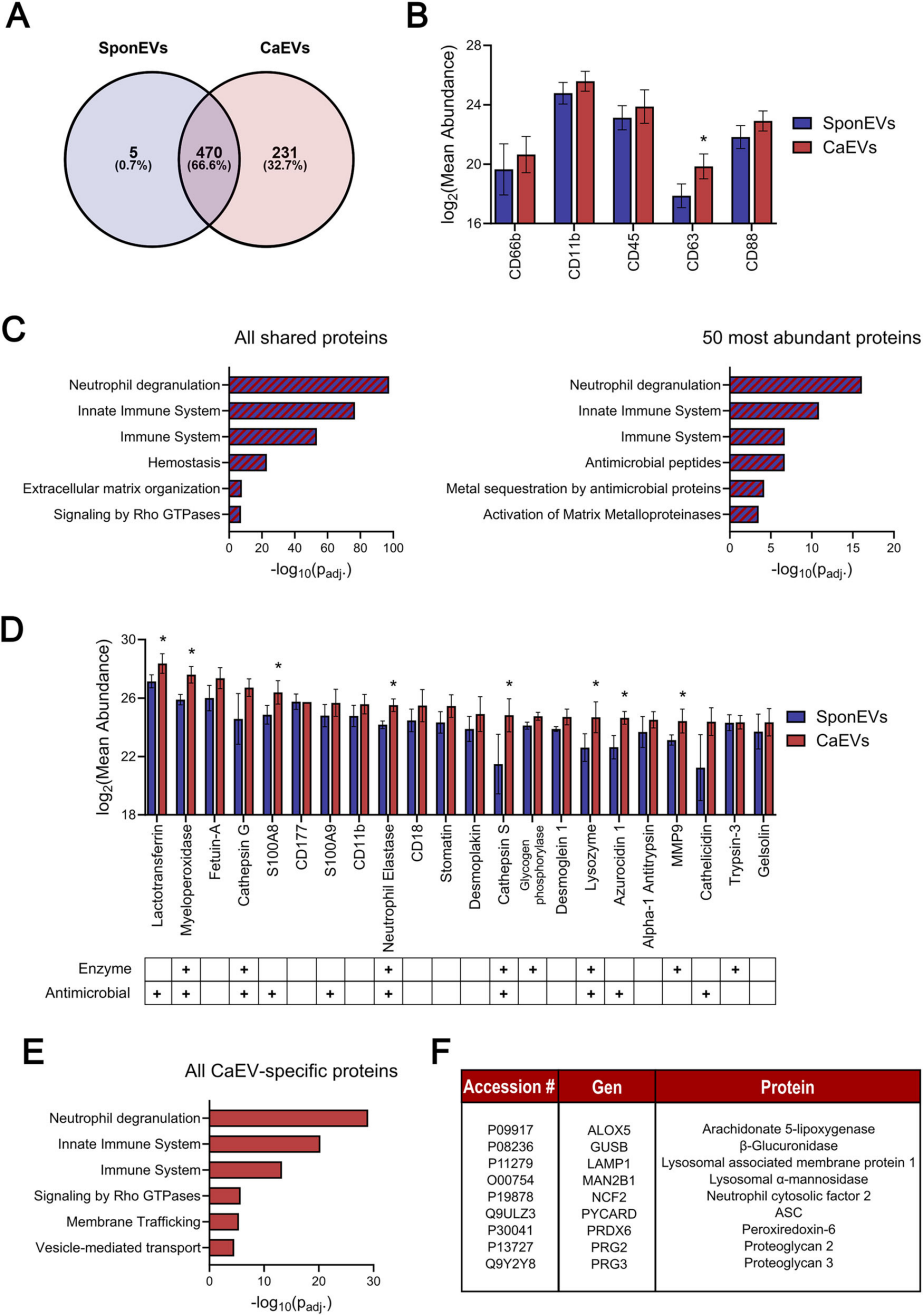
Comparative proteomic and pathway analysis reveals shared and CaEV‐specific antimicrobial signatures in neutrophil‐derived EVs. EVs were isolated from neutrophils confronted with opsonized *C. albicans* (CaEVs) or mock‐infected neutrophils (SponEVs) after 60 min of incubation. (A) Analysis of the EV proteome by LC‐MS/MS showing shared and distinct proteins in CaEVs and SponEVs that were identified in at least three out of four biological replicates. Venn diagram created with Venny 2.1 (https://bioinfogp.cnb.csic.es/tools/venny/). (B) Bars indicate the log_2_(Mean Abundance) and log_2_(Standard Deviation, SD) of neutrophil markers in SponEVs (blue) and CaEVs (red) that were analyzed for their deposition on extracellular *C. albicans* cells during interaction with neutrophils. (C) Pathway enrichment analysis of all proteins (470 proteins; left) and of the 50 most abundant proteins (right) identified in at least three out of four biological replicates in both SponEVs and CaEVs (Supplemental Data 1). (D) Bars indicate the log_2_(Mean Abundance) and log_2_(SD) of 22 proteins associated with the REACTOME pathway ‘Neutrophil degranulation’, identified as the top hit in the pathway analysis of the 50 most abundant proteins in SponEVs (blue) and CaEVs (red). Proteins marked with ‘+’ possess enzymatic and/or antimicrobial activity. Differential protein abundance was defined as a fold change of > 2, *p*‐value < 0.05 and at least identified in 3 of 4 replicates of the sample group with the highest abundance. Statistical analysis was performed with R4.4.2 and RStudio 2024.12.0. (E) Pathway enrichment analysis of all 231 proteins identified exclusively in CaEVs and present in at least three out of four biological replicates. (F) Proteins with direct or indirect antimicrobial activity identified within the CaEV‐specific protein set.

Proteomic profiling via LC‐MS/MS identified a shared core proteome of 470 proteins between the EV populations (Figure 4A). In addition, 231 proteins were uniquely detected in CaEVs, while 5 proteins were exclusive to SponEVs, indicating that fungal challenge primarily adds and enriches protein cargo on top of a stable neutrophil EV backbone rather than giving rise to a fundamentally distinct vesicle population. Notably, the shared core proteome contained several hallmark neutrophil surface and activation markers, including CD66b (cell adhesion molecule 8, CEACAM8), CD45 (receptor‐type tyrosine‐protein phosphatase C, PTPRC), CD11b (integrin α‐M, ITGAM), and CD63 (Figure 4B, Supplemental Data 1), which were also observed on the surface of extracellular *C. albicans* during neutrophil confrontation. Their increased abundance in CaEVs may reflect the increased EV release during fungal challenge. Beyond these canonical markers, the shared proteome was enriched in additional mediators of immune adherence and cell‐pathogen interactions, including complement receptor type 1 (CR1), CD11c (Integrin α‐X, ITGAX), CD18 (Integrin *β*‐2, ITGB2), CD177 (human neutrophil antigen‐2, HNA‐2), and CD162 (P‐selectin glycoprotein ligand‐1, SELPLG) (Supplemental Data 1). Together, these molecules define an adhesion‐competent EV surface architecture that is well suited to promote interactions with microbial targets, including fungal cell walls.

Importantly, analysis of the shared proteome revealed that both spontaneous and *C. albicans*‐induced neutrophil EVs are dominated by plasma‐membrane and cortical cytoskeleton modules characteristic of ectosome shedding (Supplemental Data 1). The shared set contained extensive membrane‐cytoskeleton coupling and actin‐remodeling machinery, including talin‐1 (TLN1), kindlin‐3 (FERMT3), filamin A (FLNA), vinculin (VCL), ezrin/moesin (EZR/MSN), actin‐related protein 2/3 (ARP2/3) complex subunits, cofilin‐1 (CFL1), and profilin‐1 (PFN1). In addition, key regulators of lipid asymmetry and membrane curvature, such as phospholipid scramblase 1 (PLSCR1) and the phospholipid flippase complex comprising phospholipid‐transporting ATPase 8A1 (ATP8A1) and transmembrane protein 30A (TMEM30A), were detected. The presence of these proteins strongly supports an active, regulated plasma‐membrane budding process, indicating that both SponEVs and CaEVs largely represent ectosome‐like EVs rather than products of passive membrane fragmentation. Despite this dominant ectosomal signature, canonical exosome‐associated components, including programmed cell death 6‐interacting protein (PDCD6IP/ALIX), syntenin‐1 (SDCBP), charged multivesicular body protein 2A (CHMP2A), charged multivesicular body protein 1B (CHMP1B), and vacuolar protein sorting‐associated protein 35 (VPS35), were also present in the shared core, albeit at lower relative abundance (Supplemental Data 1). This pattern is consistent with a minor but detectable contribution of the endosomal sorting complexes required for transport (ESCRT) machinery, indicative of mixed EV biogenesis pathways.

Comprehensive pathway enrichment analysis of the shared core set revealed strong associations with immune defense and inflammatory processes (Figure 4C left, Supplemental Data 2). Moreover, it highlighted the well‐established link between EVs and the Ras homolog (Rho) family of guanosine triphosphatases (Rho GTPases), central regulators of actin dynamics, vesicle formation, and intracellular trafficking, as well as the organization of the extracellular matrix (ECM), involving structural ECM components and several key proteins in remodeling. In addition, the shared proteome exhibited a conserved antimicrobial and redox‐competent signature (Supplemental Data 1), including primary and secondary granule effectors such as myeloperoxidase (MPO), neutrophil elastase (ELANE), cathepsin G (CTSG), proteinase 3 (PRTN3), bactericidal permeability‐increasing protein (BPI), azurocidin (AZU1), cathelicidin (CAMP), lysozyme (LYZ), and lactotransferrin (LTF). Consistently, components of the nicotinamide adenine dinucleotide phosphate oxidase 2 (NOX2) oxidative burst module, including the cytochrome b‐245 alpha and beta chains, CYBA (p22^phox^) and CYBB (gp91^phox^), respectively, the neutrophil cytosolic factors 1 (NCF1, p47^phox^) and 4 (NCF4, p40^phox^), Ras‐related C3 botulinum toxin substrate 2 (RAC2), and hydrogen voltage‐gated channel 1 (HVCN1), were detected across both EV populations, indicating that EVs released under both conditions originate from activation‐competent plasma‐membrane microdomains. Notably, although these antimicrobial proteins were present in the shared core proteome, the majority of them were among the 50 most abundant CaEV proteins within this core set. Accordingly, focused analysis of the CaEV Top50 revealed a pronounced enrichment of antimicrobial peptides and enzymes, collectively defining the most significantly enriched REACTOME pathway ‘neutrophil degranulation’ (Figure 4C right and 4D, Supplemental Data 2). Interestingly, several key antimicrobial proteins were significantly enriched in CaEVs compared to SponEVs, with fold changes (FC; CaEVs/SponEVs) of 2.32 for LTF, 3.25 for MPO, 2.91 for S100 calcium‐binding protein A8 (S100A8), 2.53 for ELANE, 10.26 for cathepsin S (CTSS), 4.23 for LYZ, 4.01 for AZU1, and 2.48 for matrix metalloproteinase 9 (MMP9) (Figure 4D), further supporting an enhanced neutrophil response to fungal stimuli.

Finally, analysis of the 231 CaEV‐specific proteins revealed further enrichment of pathways that overlap with those identified in the shared core proteome (Figure 4E, Supplemental Data 2), including additional proteins with direct antimicrobial activity or immunomodulatory functions (Figure 4F, Supplemental Data 1). This indicates that CaEVs are not only more abundant but are also equipped with a specialized protein cargo tailored to combat fungal pathogens.

In summary, these data demonstrate that *C. albicans* confrontation increases both the quantity and functional specialization of neutrophil‐derived EVs. While spontaneous and infection‐induced EVs share a conserved, predominantly ectosome‐like core architecture, fungal challenge drives a pronounced quantitative and qualitative remodeling of EV cargo toward enhanced antimicrobial and inflammatory effector functions.

### 3.5 CaEV‐Associated Myeloperoxidase Is Enzymatically Active

**FIGURE 5:**
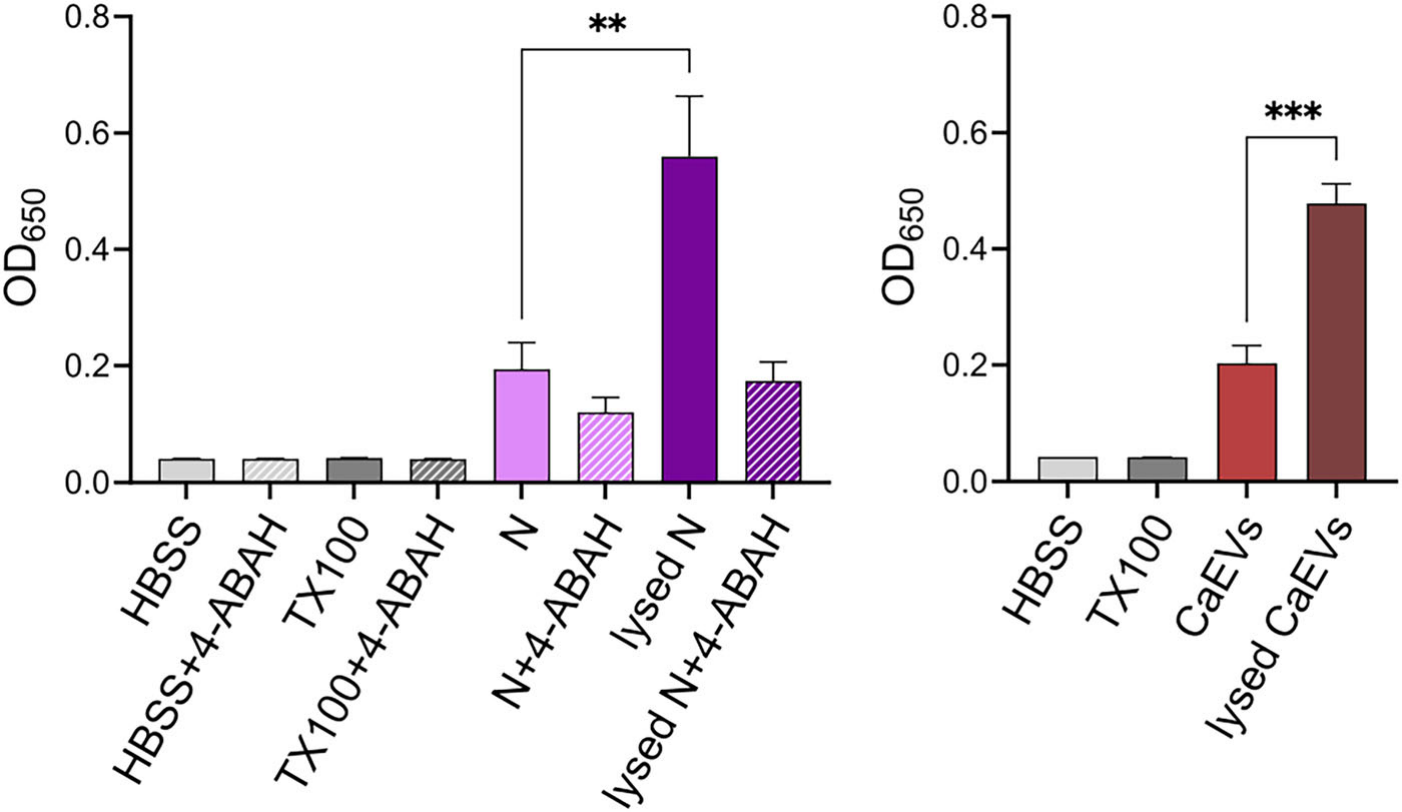
Enzymatic activity of myeloperoxidase in neutrophils and CaEVs. Myeloperoxidase (MPO) activity was determined using a colorimetric assay based on TMB oxidation. Left: MPO activity of intact neutrophils (N) or Triton X‐100‐lysed neutrophils (lysed N) in the presence or absence of the MPO inhibitor 4‐aminobenzoic acid hydrazide (4‐ABAH). HBSS and Triton X‐100 served as negative controls. Right: MPO activity of intact or Triton X‐100‐lysed CaEVs isolated from neutrophils after 60 min of confrontation with opsonized *C. albicans*. Data are presented as mean ± SD from three independent experiments using cells from different donors. Statistical analyses were performed using the two‐tailed unpaired *t*‐test, ** *p *< 0.01, *** *p *< 0.001.

To determine whether antimicrobial effector proteins carried by CaEVs retain functional enzymatic activity, we assessed activity of MPO, a representative antimicrobial enzyme, using a colorimetric assay. As expected, intact neutrophils exhibited measurable MPO activity, whereas no signal was detected in the HBSS and Triton‐X100 background controls (Figure 5 left). Cell lysis significantly increased MPO activity, consistent with improved accessibility of intracellular MPO. Furthermore, addition of the specific MPO inhibitor 4‐ABAH markedly reduced the signal in both intact and lysed neutrophils, confirming that the measured activity was MPO‐dependent (Figure 5 left). MPO activity was also detected in the CaEV preparations (Figure 5 right). While intact CaEVs exhibited measurable MPO activity above background levels, lysis of the vesicles resulted in a marked and significant increase in MPO activity. The substantially higher activity observed after membrane disruption indicates that a considerable proportion of active MPO is not readily accessible in intact CaEVs and becomes detectable only upon vesicle lysis.

### 3.6 EVs Released by Neutrophils Associate with the Surface of *C. albicans*

**FIGURE 6:**
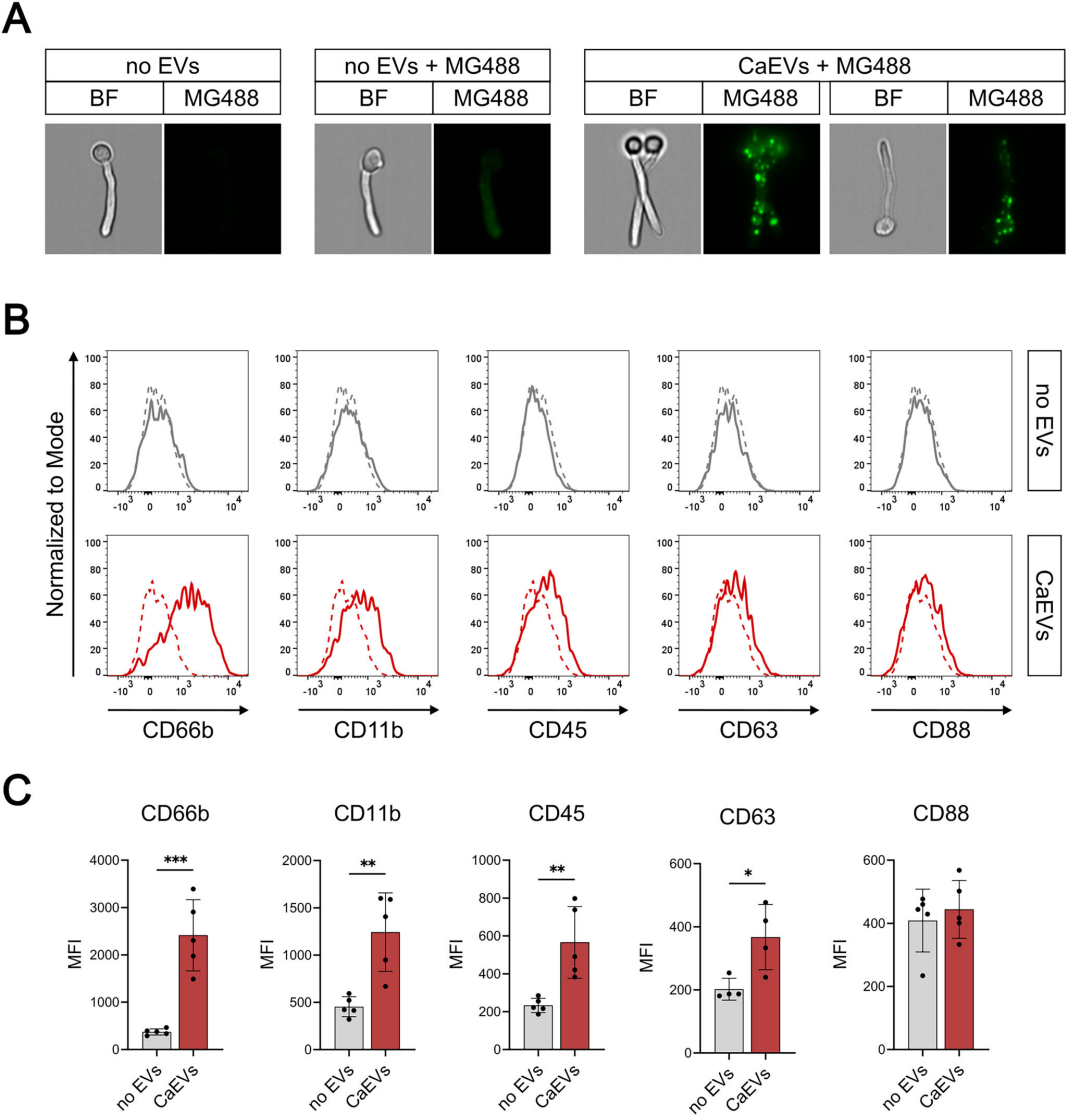
Neutrophil‐derived EVs bind to live *C. albicans* and transfer cell surface markers. EVs were isolated from the supernatants of neutrophils incubated for 60 min with opsonized *C. albicans* (CaEVs). (A) Isolated CaEVs or HBSS alone (dye‐only control) were stained with MemGlow 488 (MG488), subjected to the identical centrifugation and washing procedure to remove unbound dye, and subsequently incubated with opsonized *C. albicans* cells for 60 min. These preparations are referred to as ‘CaEVs + MG488’ and ‘no EVs + MG488’, respectively. *C. albicans* incubated in medium without EVs or MG488 served as an additional negative control (no EVs). Fluorescence associated with fungal cells was analyzed using imaging flow cytometry. Images are representative of three independent experiments (BF—Bright Field). (B) Isolated CaEVs were incubated with opsonized BFP^+^ *C. albicans* cells for 60 min. *C. albicans* cells incubated in medium without EVs served as a control. Afterwards, the fungal cells were stained and analyzed by flow cytometry. Histograms show isotype control (dashed line) and antibody staining (solid line) for *C. albicans* incubated without EVs (grey) or with CaEVs (red) and are representative of at least four independent experiments using isolated cells from different donors. (C) Quantitative analysis of CD66b, CD11b, CD45, CD63 and CD88 on *C. albicans* cells, based on median fluorescence intensity (MFI, mean ± SD from *n* = 5 experiments). Statistical analysis was performed using the two‐tailed unpaired *t*‐test, * *p* < 0.05, ** *p* < 0.01, *** *p* < 0.001.

To test our hypothesis that EVs released from neutrophils bind to *C. albicans*, we incubated opsonized fungal cells for 60 min with EVs isolated from supernatants of *C. albicans*‐challenged neutrophils and subsequently stained with MemGlow 488 (MG488), a fluorogenic membrane probe that integrates into lipid bilayers. Excess dye was removed by centrifugation and washing prior to incubation with fungal cells. In parallel, MG488 was processed identically in the absence of EVs and subjected to the same washing procedure to control for nonspecific staining. Imaging flow cytometry revealed distinct punctate MG488‐positive structures on fungal cells incubated with labeled EVs, whereas no fluorescence was detected in samples without EVs (Figure 6A). The dye‐only control exhibited a weak residual signal that localized predominantly to the growing hyphal tip, indicating limited direct association of the membrane probe with the fungal surface (Figure 6A). Importantly, this staining pattern clearly differed from the discrete punctate structures observed in the presence of EVs, supporting a direct interaction between neutrophil‐derived EVs and *C. albicans* cells. Independent visualization by laser scanning confocal microscopy of Z‐stack acquisitions, processed as maximum intensity projections, further confirmed the presence of discrete MG488‐positive puncta associated with the fungal surface, providing additional support for EV binding to *C. albicans* (Figure S5).

To further confirm EV‐fungus interaction and evaluate the presence of neutrophil proteins, flow cytometry was performed using antibodies targeting CD66b, CD45, CD11b, CD63, and CD88. Opsonized *C. albicans* cells were incubated with CaEVs for 60 min and compared to fungal cells incubated in medium without EVs (no EVs control). Notably, opsonized fungal cells did not exhibit detectable staining above isotype control levels for any of the analyzed neutrophil‐associated markers in the absence of isolated EVs (Figure 6B). In contrast, exposure to CaEVs resulted in a significant increase in the surface expression of multiple neutrophil markers on fungal cells (Figure 6B,C). For instance, the neutrophil‐specific antigen CD66b was readily detectable following EV incubation (*p* < 0.001), strongly supporting the transfer of host‐derived molecules via EVs. Consistent with results of the neutrophil confrontation (Figure 2A,B), the antigen CD88 remained undetectable on the fungal surface by flow cytometry, despite its confirmed presence in EVs by proteomic analysis (Figure 4B, Supplemental Data 1). This suggests that CD88 may either be inaccessible or unstable upon EV binding to the fungus, or alternatively, that it is not present in the EV subset that interacts with *C. albicans*. Taken together, these data strongly support the concept that neutrophil‐derived EVs bind to *C. albicans*, thereby delivering host‐derived cell surface molecules to the fungal surface, where they may potentially influence host‐pathogen interactions.

### 3.7 Complement Opsonization Promotes Binding of EVs to *C. albicans*

**FIGURE 7:**
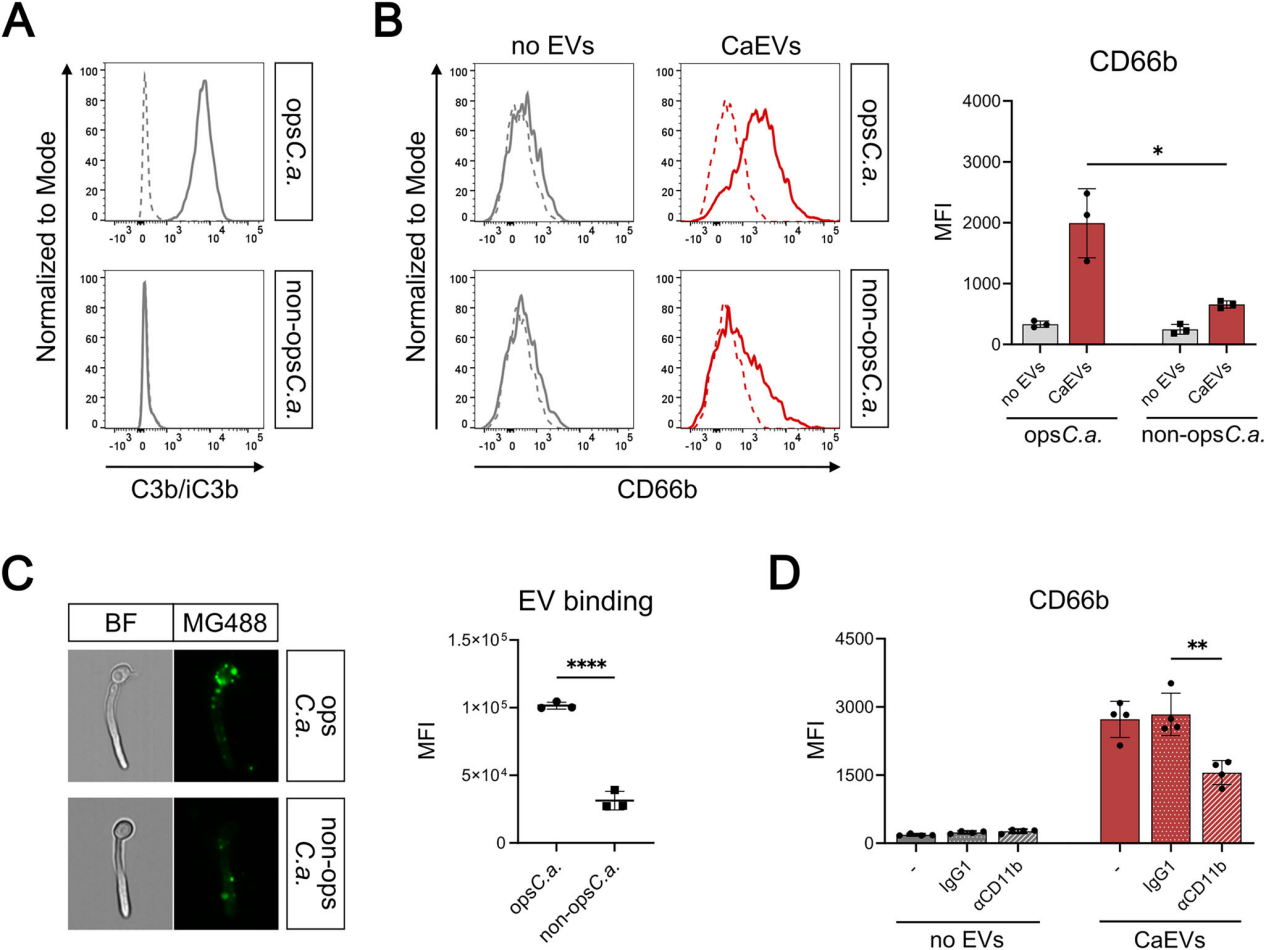
Complement‐dependent binding of neutrophil‐derived EVs to *C. albicans*. (A) *C. albicans* cells were either pre‐opsonized with autologous human serum (ops*C.a*.) or left non‐opsonized (non‐ops*C.a*.). Surface staining for C3b/iC3b was analyzed by flow cytometry. Representative histograms show isotype control (dashed line) and antibody staining (solid line). Ops*C.a*. cells show a clear shift in fluorescence intensity compared to non‐opsonized cells, confirming successful complement deposition. (B) Isolated CaEVs were incubated with opsonized and non‐opsonized *C. albicans* cells for 60 min. *C. albicans* cells incubated in medium without EVs served as a control (no EVs). Afterwards, the fungal cells were stained and analyzed by flow cytometry. Histograms (left) show isotype control (dashed line) and antibody staining (solid line) for *C. albicans* incubated without EVs (no EVs, grey) or with CaEVs (red) and are representative of three independent experiments using isolated cells from different donors. Quantitative analysis of CD66b on *C. albicans* cells (right), based on median fluorescence intensity (MFI, mean ± SD). (C) Isolated CaEVs were stained with MemGlow 488 (MG488) and incubated with opsonized and non‐opsonized *C. albicans* cells for 60 min. Binding of EVs to fungal cells was analyzed using imaging flow cytometry. Images are representative of three independent experiments (left, BF—Bright Field). Quantitative analysis of MG488 on *C. albicans* cells, based on MFI (right, mean ± SD). (D) EVs were isolated from the supernatants of neutrophils incubated for 60 min with *C. albicans* (CaEVs). Isolated CaEVs and medium without EVs as control (no EVs) were either non‐treated or pre‐incubated with anti‐CD11b antibody or the appropriate isotype control (IgG1) before incubation with opsonized BFP^+^ *C. albicans* for 60 min. Afterwards fungal cells were stained and analyzed by flow cytometry. Quantitative analysis of transferred CD66b was conducted from four independent experiments using isolated cells from different donors, based on MFI (mean ± SD). Statistical analyses for B, C and D were performed using the two‐tailed unpaired *t*‐test, * *p* < 0.05, ** *p *< 0.01, **** *p* < 0.0001.

*C. albicans* in blood triggers rapid complement activation, resulting in surface deposition of opsonins such as C3b/iC3b, thereby facilitating immune recognition, uptake and clearance (Hünniger et al. 2015). To test whether C3b/iC3b is required for EV binding, *C. albicans* cells were either pre‐opsonized or left non‐opsonized (Figure 7A), and the association of EVs with fungal cells was subsequently compared between the two conditions (Figure 7B,C). Both the binding of MG488‐labeled EVs, as assessed by imaging flow cytometry, and the detection of CD66b on the fungal surface by flow cytometry, showed significantly reduced levels for non‐opsonized *C. albicans* cells, indicating that neutrophil‐derived EVs bind preferentially to C3b/iC3b positive fungal cells.

Because some experiments in this study employed the filamentation‐defective *cph1Δ/efg1Δ* mutant, we next examined whether its altered morphology and surface properties influence complement deposition or EV association. Imaging flow cytometry revealed comparable C3b/iC3b deposition on serum‐opsonized wild‐type *C. albicans* and the *cph1Δ/efg1Δ* mutant (Figure S6A). Likewise, MG488‐labelled neutrophil‐derived EVs associated efficiently with both strains (Figure S6B), demonstrating that EV binding is independent of fungal filamentation and is not substantially affected by the altered surface characteristics of the non‐filamenting mutant.

Having established that EV binding depends primarily on complement opsonization rather than on fungal morphology, we next investigated the underlying molecular mechanisms. Given the central role of complement receptor 3 (CR3) in neutrophil recognition of opsonized pathogens, and the identification of its subunits CD11b and CD18 as abundant components of CaEVs (Figure 4D, Supplemental Data 1), we next investigated whether CD11b contributes to EV binding to *C. albicans*. Therefore, we pre‐incubated isolated CaEVs with a CD11b‐specific neutralizing antibody prior to their exposure to opsonized *C. albicans*. This treatment led to a significant reduction in the CD66b signal on the fungal surface compared to IgG1‐treated and untreated controls, indicating that EV binding to opsonized *C. albicans* is at least partially mediated via CD11b (Figure 7D).

### 3.8 EVs Reduce Phagocytosis of *C. albicans* by Neutrophils

**FIGURE 8:**
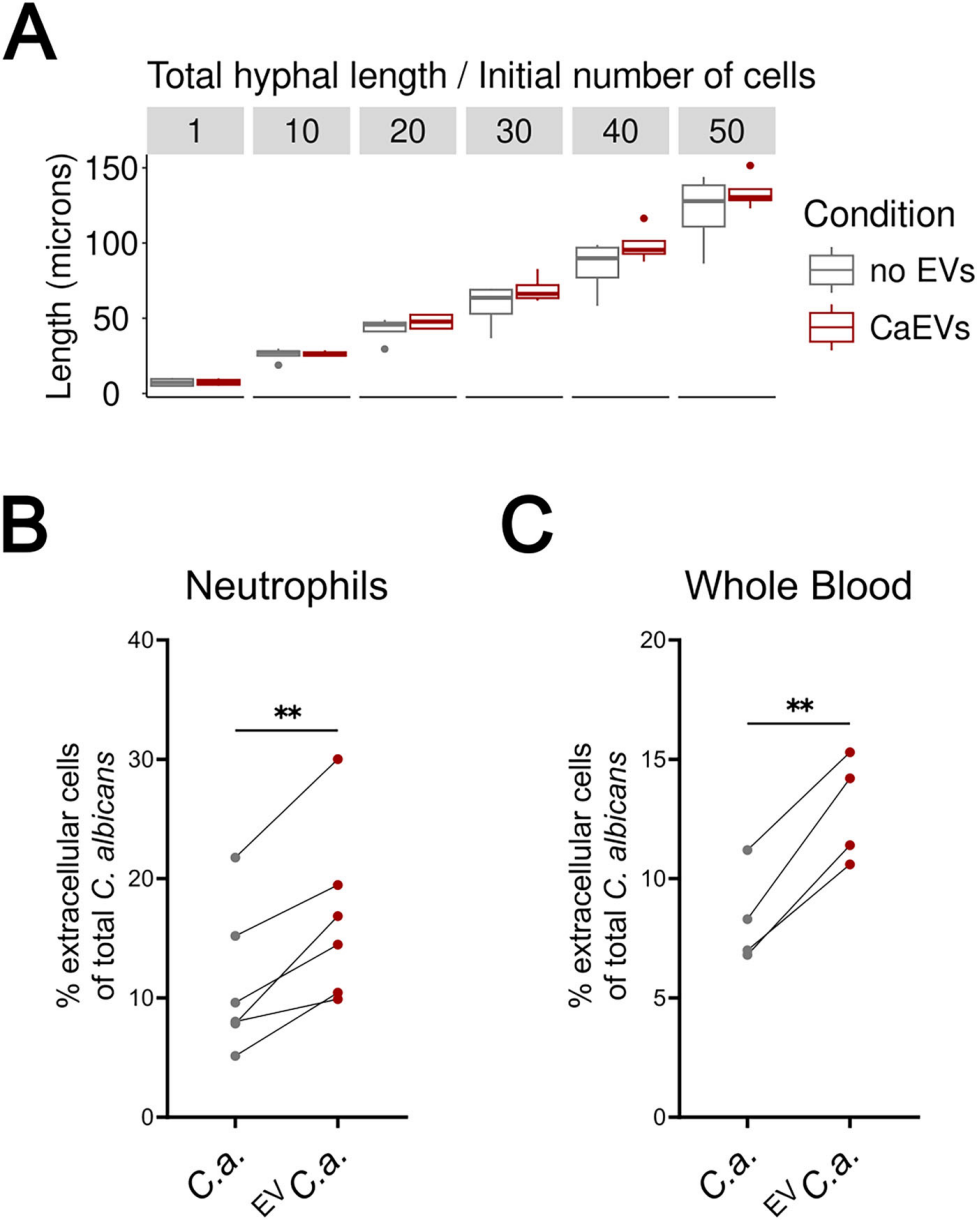
Impact of EVs on the growth and phagocytosis of *C. albicans*. EVs were isolated from the supernatants of neutrophils incubated for 60 min with opsonized *C. albicans* (CaEVs). (A) Live cell imaging of opsonized *C. albicans* incubated with CaEVs or without EVs (no EVs) was performed to analyze fungal growth by measuring hyphal length. Numbers indicate the respective time frame. Average data of five individual experiments. (B) Percentage of *C. albicans* cells remaining unphagocytosed (extracellular) after confrontation with neutrophils. Opsonized, thimerosal‐killed BFP^+^ *C. albicans* were preincubated with CaEVs (^EV^ *C.a*.) or in medium without EVs as control (*C.a*.) and then confronted with isolated neutrophils. Uptake of fungal cells was measured by flow cytometry (*n* = 6 individual experiments). (C) Phagocytosis of opsonized, thimerosal‐killed BFP^+^ *C. albicans* in whole blood after pre‐treatment with CaEVs (^EV^ *C.a*.) was compared to phagocytosis of *C. albicans* cells incubated in medium without EVs (*C.a*.). The percentage of BFP^+^ *C. albicans* cells remaining extracellular was measured by flow cytometry (*n* = 4 individual experiments). Statistical analyses for B and C were performed using the two‐tailed paired *t*‐test. ** *p* < 0.01.

Our data so far clearly demonstrate that (i) *C. albicans* enhances EV release from human neutrophils and that (ii) these EVs can bind to *C. albicans* cells, preferentially via C3b/iC3b—CR3 interaction. To investigate how this affects *C. albicans*, we performed live‐cell imaging using laser scanning microscopy to monitor hyphal growth of opsonized *C. albicans* incubated with CaEVs or in medium alone (no EVs). No differences in hyphal growth were observed between EV‐treated and control cells (Figure 8A, Figure S7), suggesting that neutrophil‐derived EVs do not impair fungal viability. Next, we evaluated whether the deposition of neutrophil‐derived EVs contributes to the reduced uptake by phagocytosis and maintenance of an extracellular *C. albicans* population in whole blood and during confrontation with purified neutrophils. For this, we employed inactivated fungal cells, since live *C. albicans* formed extensive hyphae over the course of the assay, interfering with accurate analysis. First, we confirmed that neutrophil‐derived proteins were also delivered to thimerosal‐killed *C. albicans* by isolated EVs, yielding results comparable to those obtained with live fungi (Figure S8). To assess the effect of EVs on phagocytosis, opsonized *C. albicans* cells were pre‐incubated either with neutrophil‐derived EVs (^EV^ *C.a.)* or in medium without EVs (*C.a*.) prior to confrontation with freshly isolated neutrophils from the same donor. Pre‐treatment with CaEVs led to an approximately 60% increase in the extracellular *C. albicans* population following confrontation with neutrophils, compared to pre‐incubation in medium alone (*p* < 0.01) (Figure 8B), suggesting that EV binding significantly reduces phagocytosis of *C. albicans*. Similar results were observed in freshly drawn whole blood from the same donors, with a significantly higher percentage of extracellular ^EV^ *C.a*. cells compared to controls (*p* < 0.01) (Figure 8C). In line with the findings from purified neutrophils, the proportion of extracellular fungal cells in blood was 56.7% ± 15.9% higher for ^EV^ *C.a*. compared to untreated control cells (*C.a*.). Taken together, these results reveal a previously unknown role for neutrophil‐derived EVs in reducing the susceptibility of *C. albicans* to phagocytic uptake.

**FIGURE 9:**
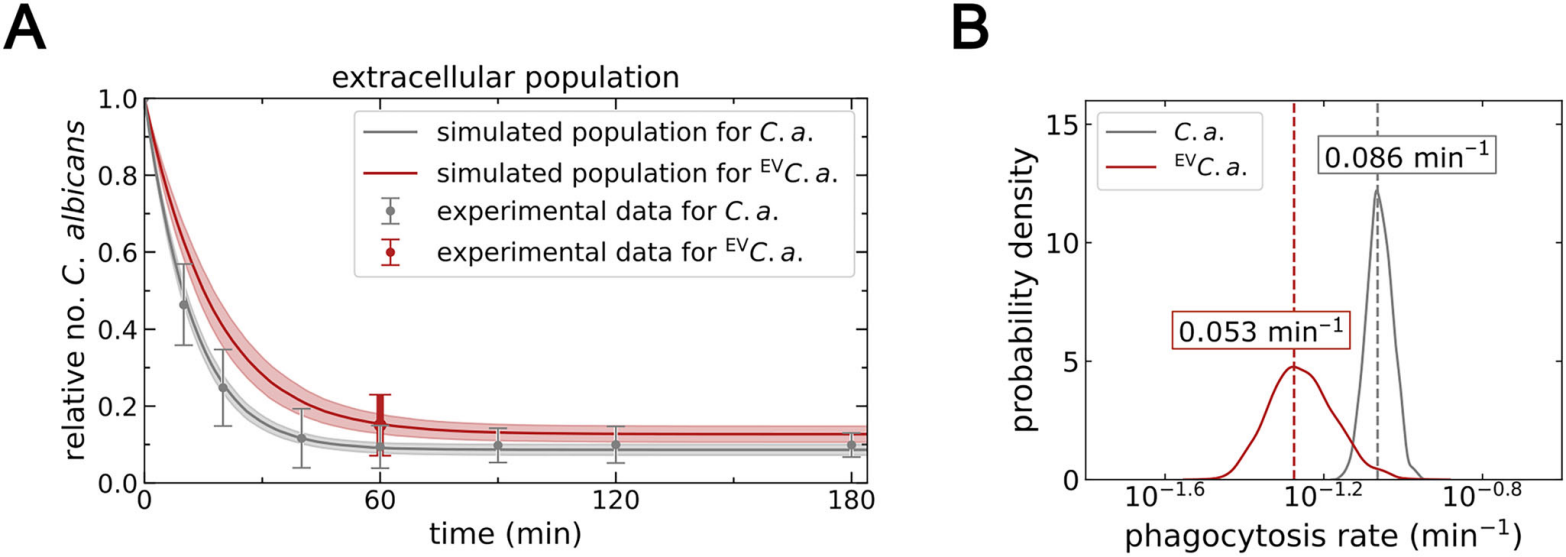
Fitting results of the state‐based model to experimental data. Model fitting and parameter inference were carried out using Approximate Bayesian Computation (ABC). (A) Simulated extracellular population dynamics of the EV‐decorated (^EV^ *C.a*., red) and untreated (*C.a*., grey) *C. albicans* overlaid with experimental measurements. The solid lines show the mean, and shaded areas the standard deviation over 100 simulations with parameter sets sampled from the inferred posterior. (B) Marginal posterior distributions of phagocytosis rates by neutrophils of ^EV^ *C.a*. and *C.a*. Dashed lines indicate the mode of the posterior distribution, and the corresponding values of the mode are shown within the boxes above the marginal posteriors.

To quantify the impact of EV deposition on phagocytosis of *C. albicans*, we employed a previously established virtual infection model (Hünniger et al. 2014). In this model, we introduced a distinct phagocytosis rate for the EV‐decorated population of *C. albicans* and applied Approximate Bayesian Computation (ABC) for parameter inference. Model fitting to the experimental data was performed by averaging over 100 simulations using parameter sets randomly sampled from the inferred posterior distribution (Figure 9A, Figure S9A). The joint posterior mode was then used to quantify the model parameters (Figure 9B, Figure S9B), revealing a clear difference in the phagocytosis rates by neutrophils of untreated control cells (*C.a*.) and EV‐decorated *C. albicans* (^EV^ *C.a*.), with $\phi_{N}$(*C.a*.) = 0.086 min^−1^ and $\phi_{N}$(^EV^ *C.a*.) = 0.053 min^−1^, respectively. This 1.6‐fold reduction in the estimated phagocytosis rate for the EV‐decorated fungal cells quantitatively supports the role of neutrophil‐derived EVs in decreasing the susceptibility of *C. albicans* to phagocytosis.

## 4 Discussion

In this study, we uncover a previously unrecognized, host‐driven mechanism that reduces phagocytic clearance and promotes the maintenance of an extracellular *C. albicans* population during fungal bloodstream infection. Deposition of neutrophil‐derived EVs onto the fungal surface impairs immune recognition of *C. albicans*. Reducing phagocytic uptake then promotes the persistence of viable extracellular fungal cells in human blood. Unlike classical fungal immune‐evasion strategies, this mechanism solely originates from the host response. This introduces a new dimension to host‐pathogen interactions in *C. albicans* bloodstream infection and is potentially relevant in the context of *C. albicans* dissemination to secondary sites of infection.

The commensal fungus *C. albicans* is one of the leading causes of bloodstream infections, particularly in immunocompromised patients, due to its ability to disseminate from colonized tissues and to become invasive (Pappas et al. 2018). While various types of phagocytes contribute to innate immunity against fungi, neutrophils represent the primary effector cells responsible for the uptake of *C. albicans* and play a crucial role in the clearance of fungal cells from the bloodstream. However, our previous studies have shown that, despite the rapid phagocytic uptake of the majority of *C. albicans* cells by neutrophils and monocytes, a subpopulation remains extracellular in human blood for extended periods (Hünniger et al. 2014; Lehnert et al. 2021; Lehnert et al. 2015). The mechanisms underlying this phenomenon have remained largely unknown. Here, we demonstrate that this form of immune escape is not related to established virulence or immune‐evasion traits, but instead depends on activation of the host response.

A striking observation was the time‐dependent appearance of canonical neutrophil surface markers, including CD66b, CD11b, CD45, and CD63, together with Annexin V binding on non‐phagocytosed fungal cells. This pattern suggested that host‐derived membrane structures accumulate on the fungal surface during neutrophil confrontation. We therefore hypothesized that neutrophil‐derived EVs bind to *C. albicans* and mediate this surface remodeling. EVs are small bilayered membrane particles released by most cell types (Buzas 2023). In neutrophils, EV release can occur spontaneously or in response to stimulation, contributing to the antimicrobial defense as well as immune regulation (Alvarez‐Jimenez et al. 2018; Lorincz et al. 2015; Shrestha and Hong 2023).

Following MISEV2023 guidelines, we characterized EVs using nanoparticle tracking analysis (NTA), cryogenic transmission electron microscopy (cryo‐TEM), and protein marker analysis, allowing assessment of particle size distribution, vesicle morphology, and EV‐associated marker expression (Welsh et al. 2024). The size distribution of our EV preparations was comparable to that reported for previously described neutrophil‐derived EVs, including those released during fungal infections (Gomez et al. 2020; Kolonics et al. 2021; Rafiq et al. 2022). Previous studies have shown that EVs carry diverse bioactive molecules, including proteins, nucleic acids, and enzymes, that facilitate intercellular communication by transferring host components to target cells (Brakhage et al. 2021). In line with this, we detected the neutrophil‐derived membrane proteins CD66b, CD11b, CD45, and CD63 on non‐phagocytosed fungal cells following exposure to neutrophils. The same proteins were identified in purified EVs by mass spectrometry, consistent with previous reports (Dalli et al. 2013; Gasser et al. 2003). Western blot analysis further confirmed the presence of the EV‐associated proteins CD11b, CD63, and Annexin A1 in these preparations. Incubation of opsonized *C. albicans* with isolated neutrophil‐derived EVs reproduced the appearance of these markers, indicating that they are EV‐associated and become exposed upon vesicle binding. Together, these findings indicate that neutrophils release EVs that bind to the fungal surface, potentially masking the pathogen and thereby reducing its susceptibility to uptake by phagocytosis.

Mechanistically, EV binding occurred preferentially on *C. albicans* cells that had been opsonized with complement components C3b/iC3b. Several *C. albicans* surface molecules have been implicated in complement interactions. Earlier studies described a CR3‐related protein (CR3‐RP) and additional putative complement receptor analogs that may interact with iC3b, although their molecular identities and physiological relevance remain incompletely resolved (Alaei et al. 1993; Bujdakova et al. 2010). In addition, well‐characterized proteins such as Pra1, Gpm1, Gpd2, and Hgt1 recruit host complement regulators and thereby modulate local C3 fragment deposition (Singh et al. 2020). These fungal factors may collectively influence EV accumulation on complement‐opsonized fungal cells. On the host side, our proteomic analyses revealed the presence of several complement receptors on neutrophil‐derived EVs, including CR1, CD11b/CD18 (CR3), and CD11c/CD18 (CR4), which may facilitate interactions with opsonized fungal cells. This interpretation is supported by previous reports describing complement receptors in neutrophil‐derived microparticles (Hess et al. 1999; Pluskota et al. 2008) and by studies demonstrating the role of CR3 in recognizing C3b/iC3b‐opsonized *C. albicans* (Hünniger et al. 2015). Consistently, blocking CD11b significantly reduced EV binding, further supporting the involvement of CR3. EVs may also interact with opsonized pathogens via CR1 (Hess et al. 1999), while the detection of CR4 in our EV proteome suggests an additional link to the complement system, as CR4, like CR3, binds iC3b (Lukacsi et al. 2017). Interestingly, although complement deposition normally promotes fungal clearance by enhancing phagocytosis, our data indicate that it may also facilitate EV accumulation on the fungal surface. This dual role highlights the complex interplay between host defense mechanisms and pathogen persistence during bloodstream infection. Notably, EVs also associated with non‐opsonized *C. albicans* to a lesser extent, suggesting that fungal surface molecules additionally contribute to EV attachment. In this context, CR3 may play a dual role, as it is capable of recognizing both complement components and certain fungal PAMPs (O'Brien et al. 2012; van Bruggen et al. 2009), indicating that multiple receptor‐ligand interactions likely contribute to EV‐fungus binding. Moreover, the detection of Toll‐like receptor 2 (TLR2) in the EV proteome raises the possibility that direct recognition of fungal cell‐wall components, particularly phospholipomannans, contributes to EV association with *C. albicans* (Jouault et al. 2003). However, the relative contribution of TLR2 to EV binding remains to be determined.

Proteomic analysis revealed that *C. albicans*‐induced EVs were enriched in numerous antimicrobial proteins derived from neutrophil granules, including neutrophil elastase, myeloperoxidase, cathepsin G, azurocidin, lactotransferrin, and cathelicidin. Such enrichment is consistent with infection‐induced EVs described during interactions with *Aspergillus fumigatus* (Shopova et al. 2020). In addition, the EV proteome contained multiple proteins involved in membrane‐cytoskeleton coupling and vesicle trafficking, supporting the notion that CaEVs are generated through active vesicle shedding rather than passive release from damaged cells. Together with the minimal neutrophil apoptosis observed under our experimental conditions, these findings argue against a major contribution of apoptosis‐derived vesicles, including apoptotic bodies, to fungal surface coating. Likewise, a role for NET‐associated extracellular material during neutrophil confrontation cannot be excluded. Nevertheless, the fungal surface phenotype was reproduced using isolated EV preparations, as demonstrated by the transfer of EV‐associated neutrophil markers to *C. albicans* in the absence of neutrophils and the formation of discrete punctate fluorescent structures on the fungal surface by labeled EVs. Together, these findings support neutrophil‐derived EVs as the principal mediators of the observed surface decoration. Given the complex cargo of CaEVs, it is conceivable that these vesicles exert additional immunomodulatory effects. In line with this possibility, EVs released by *C. albicans*‐infected monocytes have been shown to carry immunoregulatory mediators such as TGF‐β1 (Halder et al. 2020).

Importantly, and unlike *A. fumigatus*‐induced neutrophil EVs or EVs derived from a human oral mucosal epithelial cell line, CaEVs did not inhibit fungal growth or hyphal elongation under the conditions tested (Shopova et al. 2020; Zhao et al. 2022). Although MPO retained enzymatic activity in CaEVs, the marked increase in activity following vesicle lysis suggests that much of the active enzyme is not readily accessible in intact vesicles, which could explain the lack of detectable antifungal activity of CaEVs. Despite this absence of direct antifungal activity, the interaction of CaEVs with *C. albicans* significantly modulated host‐pathogen interactions by altering phagocytosis by neutrophils. Pre‐incubation of *C. albicans* with neutrophil‐derived EVs reduced fungal uptake by neutrophils both in purified cell systems and in whole blood. This experimentally observed effect was supported by mathematical modeling, which quantified a 1.6‐fold decrease in the phagocytosis rate of EV‐decorated compared to untreated *C. albicans*. It is important to note that fungal cells in the control condition were not entirely devoid of EV exposure, as neutrophils continuously release EVs upon contact with *C. albicans*. Consequently, even the untreated fungal cells likely acquired host‐derived EVs, suggesting that the observed differences reflect different degrees of EV binding rather than a strict comparison between EV‐decorated and non‐EV‐decorated cells. These findings indicate that surface decoration of *C. albicans* with host‐derived EVs contributes to reduced phagocytic uptake and the maintenance of an extracellular fungal population. One plausible explanation is a steric masking effect, whereby EV deposition limits the accessibility of fungal pathogen‐associated molecular patterns as well as complement‐derived opsonins such as C3b/iC3b, thereby reducing recognition by phagocytes. In addition, the transfer of host‐derived membrane proteins to the fungal surface may generate a host‐like molecular signature that impairs the discrimination between self and non‐self. Although our proteomic analyses did not identify specific host‐derived proteins expected to directly inhibit neutrophil activation, the presence of neutrophil‐derived surface components may nevertheless influence phagocyte recognition and contribute to reduced fungal uptake. Moreover, our findings argue against a major contribution of active fungal surface remodeling to the reduced phagocytic uptake, as EV association was largely independent of fungal viability and filamentation. Given the central role of phagocytosis in early antifungal defense, EV‐mediated ‘masking’ could contribute to the persistence of an extracellular subpopulation and potentially facilitate fungal dissemination during bloodstream infection. These findings therefore highlight that a physiological component of the innate immune response may, under certain conditions, paradoxically favor fungal persistence rather than host protection.

Several limitations should be noted. This study focused on neutrophil‐derived EVs, while EVs from other blood cells such as monocytes, platelets, or endothelial cells may also interact with *C. albicans* and modulate phagocytosis *in vivo*. Furthermore, it remains to be determined whether EV‐mediated surface decoration is specific to *C. albicans* or represents a more general mechanism affecting other extracellular pathogens in the bloodstream, including additional fungi and bacteria. The *ex vivo* models used here, although highly informative, do not fully capture the spatial and temporal complexity of infection within tissues. Finally, although we identified complement‐dependent EV binding, the precise molecular mechanisms remain to be elucidated.

In summary, this study identifies a previously unrecognized host‐driven mechanism in which neutrophil‐derived EVs bind to complement‐opsonized *C. albicans*, thereby reducing susceptibility to phagocytic uptake. Together with previous evidence that neutrophil EVs influence complement activation and both innate and adaptive immune responses (Hsu et al. 2025; Kalluri 2024), our findings expand the functional repertoire of neutrophil EVs, revealing a host‐derived mechanism that modulates immune recognition and promotes fungal persistence in the bloodstream.

## Author Contributions

**Jennifer J. Patitz**: investigation, methodology, visualization, writing – original draft, formal analysis. **Natalie E. Nieuwenhuizen**: formal analysis, investigation, methodology, supervision, writing – original draft. **Anastasia Solomatina**: formal analysis, software, writing – review and editing. **Yann Bachelot**: formal analysis, software, writing – review and editing. **Thomas Krüger**: data curation, formal analysis, investigation, writing – review and editing. **Carl‐Magnus Svensson**: formal analysis, software, writing – review and editing. **Stephanie Hoeppener**: data curation, investigation, writing – review and editing. **Ann‐Kathrin Zimmermann**: data curation, investigation, writing – review and editing. **Matthew G. Blango**: data curation, investigation, writing – review and editing. **Ronny Martin**: resources, writing – review and editing. **Olaf Kniemeyer**: resources, writing – review and editing. **Theresa Lange**: resources, writing – review and editing. **Axel A. Brakhage**: project administration, resources, writing – review and editing. **Marc Thilo Figge**: conceptualization, formal analysis, funding acquisition, project administration, resources, supervision, writing – review and editing. **Oliver Kurzai**: conceptualization, funding acquisition, project administration, supervision, writing – review and editing, resources. **Kerstin Hünniger‐ast**: conceptualization, formal analysis, investigation, methodology, supervision, visualization, writing – original draft.

## Funding

The study was supported by the Deutsche Forschungsgemeinschaft (DFG) within the Collaborative Research Center CRC TR124 FungiNet ‘Pathogenic fungi and their human host: Networks of interaction’, DFG project number 210879364 (project A1 to A.A.B., project B4 to M.T.F., project C3 to O.Ku., project Z2 to O.Kn.), and by the DFG Collaborative Research Center SFB 1583/1 ‘DECIDE’ (project number 492620490, project C3 to O.Ku.). Further support was provided by the German Federal Ministry of Education and Research (BMBF) through the CompLS—Computational Life Sciences program within the project MuMoSim (project 031L0291A to M.T.F., project 031L0291B to O.Ku.), and through the funding program Photonics Research Germany, project Leibniz Center for Photonics in Infection Research (subproject LPI‐BT3, contract number 13 N15709 to M.T.F.). The project FKZ 01K12012 ‘RFIN—RNA Biology of Fungal Infections’ (to M.G.B.) was funded by the German Federal Ministry for Research, Technology and Space (BMFTR). T.L. was funded by the DFG Priority Program SPP2225 ‘Exit strategies of intracellular pathogens’ (project number 446404928). J.J.P. was funded by the European Union's Horizon 2020 research and innovation program under grant agreement 847507 (HDM‐FUN).

The funders had no role in study design, data collection and analysis, decision to publish, or preparation of the manuscript.

## Conflicts of Interest

The authors declare no conflicts of interest.

## Supporting information




**Supporting Information**: jev270363‐sup‐0001‐FigureS1‐S9.docx

**Supporting Information**: jev270363‐sup‐0002‐SuppMat.xlsx

**Supporting Information**: jev270363‐sup‐0003‐SuppMat.xlsx

## Data Availability

The mass spectrometry proteomics data have been deposited at the ProteomeXchange Consortium via the PRIDE partner repository with the dataset identifier PXD070460 and https://doi.org/10.6019/PXD070460.
