# Supplementary material for: Host‐to‐Pathogen Transfer of Neutrophil Components via Extracellular Vesicles Shields Candida albicans From Immune Attack in Human Blood
Source: J Extracell Vesicles. 2026 Aug 29;15(9):e70363. doi: 10.1002/jev2.70363 (PMC13525865; doi:10.1002/jev2.70363)
Supplement: Supplementary file 1 [file JEV2-15-e70363-s003.docx]

**Supporting Information**

**Host-to-Pathogen Transfer of Neutrophil Components via Extracellular Vesicles Shields *Candida albicans* from Immune Attack in Human Blood**

Jennifer J. Patitz^1^, Natalie E. Nieuwenhuizen^1^, Anastasia Solomatina^2^, Yann Bachelot^2,3^, Thomas Krüger^4^, Carl-Magnus Svensson^2^, Stephanie Hoeppener^5,6^, Ann-Kathrin Zimmermann^4^, Matthew G. Blango^7^, Ronny Martin^1^, Olaf Kniemeyer^4^, Theresa Lange^8^, Axel A. Brakhage^4,9^, Marc Thilo Figge^2,9^, Oliver Kurzai^1,10,11,*^, Kerstin Hünniger-Ast^1,10^

^1^Institute for Hygiene and Microbiology, Julius Maximilians University of Würzburg, Würzburg, Germany

^2^Department of Applied Systems Biology, Leibniz Institute for Natural Product Research and Infection Biology - Hans Knöll Institute, Jena, Germany

^3^Faculty of Biological Sciences, Friedrich Schiller University, Jena, Germany

^4^Department of Molecular and Applied Microbiology, Leibniz Institute for Natural Product Research and Infection Biology - Hans Knöll Institute, Jena, Germany

^5^Laboratory of Organic and Macromolecular Chemistry, Friedrich Schiller University, Jena, Germany

^6^Jena Center for Soft Matter, Friedrich Schiller University, Jena, Germany

^7^Junior Research Group RNA Biology of Fungal Infections, Leibniz Institute for Natural Product Research and Infection Biology - Hans Knöll Institute, Jena, Germany

^8^Department of Microbial Pathogenicity Mechanisms, Leibniz Institute for Natural Product Research and Infection Biology - Hans Knöll Institute, Jena, Germany

^9^Institute of Microbiology, Faculty of Biological Sciences, Friedrich Schiller University, Jena, Germany

^10^Research Group Fungal Septomics, Leibniz Institute for Natural Product Research and Infection Biology - Hans Knöll Institute, Jena, Germany

^11^National Reference Center for Invasive Fungal Infections (NRZMyk), Leibniz Institute for Natural Product Research and Infection Biology - Hans Knöll Institute, Jena, Germany

^*^ Corresponding author. Institute for Hygiene and Microbiology, Julius Maximilians University of Würzburg, Josef-Schneider-Str. 2 / Building E1, 97080 Würzburg, Germany. Email: oliver.kurzai@uni-wuerzburg.de

**Supplemental Data 1**

Full dataset of EV proteins identified by LC-MS/MS in *C. albicans*-induced EVs (CaEVs) and spontaneous EVs (SponEVs).

**Supplemental Data 2**

REACTOME pathway enrichment analysis of the EV proteome.

**Supplemental Figures**

**FIGURE S1**

**FIGURE S1** Gating strategy for extracellular *C. albicans*. Isolated neutrophils were confronted with opsonized BFP^+^ *C. albicans* for 60 min. After co-incubation, cells were stained with antibodies and analyzed by flow cytometry. Extracellular fungal cells were gated as BFP^+^ and low side scatter (SSC).

**FIGURE S2**

**FIGURE S2** Cell viability staining of sorted extracellular thimerosal-killed *C. albicans* cells*.* Whole blood was infected with thimerosal-killed GFP^+^ *cph1Δ/efg1Δ* mutant for 120 min followed by cell sorting of extracellular fungal cells and subsequent staining with calcofluor white (CFW) and propidium iodide (PI). All sorted extracellular thimerosal-killed *cph1Δ/efg1Δ* cells showed positive PI staining. Representative fluorescence images were taken using a Zeiss LSM 780 confocal microscope. In the merged image, GFP fluorescence appears green, CFW staining blue, and PI staining red. Scale: 20 µm. The non-filamentous *cph1Δ/efg1Δ* mutant was used for technical reasons related to cell sorting.

**FIGURE S3**

**FIGURE S3** Apoptosis Assay. After confrontation of neutrophils with opsonized *C. albicans*, the percentage of apoptotic cells was determined by staining with Annexin V and propidium iodide (PI). Only samples with ≤10 % apoptotic cells (Annexin V^+^ PI^-^) were used for EV isolation, to avoid isolation of apoptotic bodies. Annexin V^-^ PI^-^ cells and Annexin V^+^ PI^+^ cells were classified as viable and dead cells, respectively. Mean values of five individual experiments are shown. The dashed line represents 10% border.

**FIGURE S4**

**FIGURE S4** Analysis of CYCS and CANX in neutrophils and neutrophil-derived EVs. Representative immunoblot analysis of protein lysates from primary human neutrophils (N), prepared immediately after isolation, and from EVs isolated 60 min after confrontation of neutrophils with opsonized *C. albicans* (CaEVs). Membranes were probed for CYCS (cytochrome c; mitochondrial marker) and CANX (calnexin; endoplasmic reticulum marker), which served as intracellular contamination controls for EV preparations. ANXA1 (Annexin A1) was included as a positive EV marker. Molecular weight markers (M) are indicated.

**FIGURE S5**

**FIGURE S5** Confocal microscopy confirms the association of neutrophil-derived EVs with the surface of *C. albicans*. CaEVs were isolated from neutrophil-*C. albicans* confrontation assays, labeled with MemGlow™ 488 (MG488), and incubated with opsonized live *C. albicans* cells for 60 min at 37°C. EV association with fungal cells was visualized by laser scanning confocal microscopy, with representative maximum intensity projections of Z-stacks demonstrating discrete punctate fluorescent structures associated with the fungal surface, consistent with EV binding. Scale bar, 10 µm.

**FIGURE S6**

**FIGURE S6** Comparable complement deposition and EV binding to wild-type and filamentation-defective *C. albicans*. (A) Representative ImageStream images showing C3b/iC3b deposition on opsonized wild-type *C. albicans* and the *cph1Δ/efg1Δ* mutant. IgG1 controls are shown for comparison. (B) EVs were isolated from the supernatants of neutrophils incubated for 60 min with either wild-type *C. albicans* (CaEVs) or the *cph1Δ/efg1Δ* mutant (cph1Δ/efg1ΔEVs). Following isolation, EVs were stained with MemGlow 488 (MG488) and incubated with the corresponding opsonized fungal strain for 60 min. EV association with fungal cells was assessed by imaging flow cytometry. Fungal cells incubated in medium without EVs served as controls (no EVs). Images are representative of three independent experiments (BF - Bright Field).

**FIGURE S7**

**FIGURE S7** *C. albicans* growth in the presence of EVs. EVs were isolated from the supernatants of neutrophils incubated for 60 min with opsonized *C. albicans* (CaEVs). Live cell imaging of opsonized *C. albicans* incubated with CaEVs or without EVs as a control (no EVs) was performed to analyze fungal growth on a Zeiss LSM 780 confocal microscope. Images are representative of five independent experiments using isolated cells from different donors. Numbers indicate the respective time frame (1 time frame = 5 min). Scale bar, 100 µm.

**FIGURE S8**

**FIGURE S8** Neutrophil-derived EVs transfer host markers to killed *C. albicans*. EVs were isolated from neutrophils confronted with opsonized *C. albicans* (CaEVs). Subsequently, opsonized, thimerosal-killed BFP^+^ *C. albicans* cells were incubated with EVs or without EVs as a control (no EVs). After 60 min of incubation, fungal cells were stained and analyzed by flow cytometry. (A) Representative histograms of *C. albicans* incubated without EVs (grey) or with CaEVs (red) showing the isotype control (dashed line) and antibody staining (solid line). Histograms are representative of four independent experiments using isolated cells from different donors. (B) Quantitative analysis of CD66b, CD11b, CD45, CD63 and CD88 on *C. albicans* cells, based on median fluorescence intensity (MFI, mean ± SD from n = 4 experiments). Statistical analysis was performed using the two-tailed unpaired *t*-test, ** *p* < 0.01, *** *p* < 0.001, **** *p* < 0.0001.

**FIGURE S9**

**FIGURE S9** Extended fitting results of the state-based model to experimental data. (A) Simulated dynamics of the four pathogen populations - associated with neutrophils, associated with monocytes, alive, and killed - for EV-decorated (^EV^*C.a.*, red) and untreated (*C.a.,* grey) *C. albicans*, overlaid with corresponding experimental measurements. Mean and standard deviation over 100 runs are shown by the solid lines and shaded areas, respectively. (B) Marginal posterior distributions of the remaining six model parameters - extracellular killing parameters $\kappa_{EK}$ and $\gamma$, killing rates for neutrophils $\kappa_{N}$ and monocytes $\kappa_{M}$, phagocytosis rate for monocytes $\phi_{M}$, and immune evasion rate $\rho$ - with the joint posterior mode indicated by dashed lines.
